# Changes in waterfowl migration phenologies in central North America: Implications for future waterfowl conservation

**DOI:** 10.1371/journal.pone.0266785

**Published:** 2022-05-18

**Authors:** Kent Andersson, Craig A. Davis, Grant Harris, David A. Haukos

**Affiliations:** 1 Department of Natural Resource Ecology and Management, Oklahoma State University, Stillwater, Oklahoma, United States of America; 2 U.S. Fish and Wildlife Service, Albuquerque, New Mexico, United States of America; 3 U.S. Geological Survey, Kansas Cooperative Fish and Wildlife Research Unit, Kansas State University, Manhattan, Kansas, United States of America; U.S. Geological Survey, UNITED STATES

## Abstract

Globally, migration phenologies of numerous avian species have shifted over the past half-century. Despite North American waterfowl being well researched, published data on shifts in waterfowl migration phenologies remain scarce. Understanding shifts in waterfowl migration phenologies along with potential drivers is critical for guiding future conservation efforts. Therefore, we utilized historical (1955–2008) nonbreeding waterfowl survey data collected at 21 National Wildlife Refuges in the mid- to lower portion of the Central Flyway to summarize changes in spring and autumn migration phenology. We examined changes in the timing of peak abundance from survey data at monthly intervals for each refuge and species (or species group; *n* = 22) by year and site-specific temperature for spring (Jan–Mar) and autumn (Oct–Dec) migration periods. For spring (*n* = 187) and autumn (*n* = 194) data sets, 13% and 9% exhibited statistically significant changes in the timing of peak migration across years, respectively, while the corresponding numbers for increasing temperatures were 4% and 9%. During spring migration, ≥80% of significant changes in the timing of spring peak indicated advancements, while 67% of significant changes in autumn peak timing indicated delays both across years and with increasing temperatures. Four refuges showed a consistent pattern across species of advancing spring migration peaks over time. Advancements in spring peak across years became proportionally less common among species with increasing latitude, while delays in autumn peak with increasing temperature became proportionally more common. Our study represents the first comprehensive summary of changes in spring and autumn migration phenology for Central Flyway waterfowl and demonstrates significant phenological changes during the latter part of the twentieth century.

## Introduction

There has been a considerable amount of literature published in recent decades documenting changes in phenological events of plants and animals in response to climate change [1–4]. Much of this attention has been focused on shifts in avian migration phenologies [5–9], with most of the studies examining changes in the migration phenology of spring migrants [10, 11]. Based on numerous studies, it is clear that climate change is influencing migration phenologies of bird species across the globe. Moreover, it appears that the migration timing of many bird species is more affected by climate change during spring migration than autumn migration [7]. For spring migration, the dominating pattern has been that migration dates have advanced over time as global temperatures have increased, while the patterns for autumn migration have been more variable, though delays appear more common than advancements [5–9].

Each year, considerable resources are invested by federal, state, and provincial agencies as well as private organizations in the conservation and management of waterfowl in North America. Yet, to date, comparatively little data have been published on phenological shifts in waterfowl migration in general [12–14], and North American waterfowl in particular [15–19]. In fact, we found only 3 North American studies that presented data for more than 3 species of waterfowl [15, 18, 19] and just 2 studies that offered any data at all on autumn migration [15, 19]. What published data exist indicate that spring migration generally occurred earlier over time, while for autumn migration, delays in migration timing were more common than advancements. Moreover, model projections of reduced snow cover and rising air temperatures under a warming climate are expected to lead to northward shifts in wintering ranges and delayed autumn migration for several dabbling duck species in the eastern third of North America [20]. Further, the rate of change in migratory passage by year or temperature may vary with latitude [8, 21].

The current lack of published data on basic changes in migration phenology for North American waterfowl is problematic considering that successful waterfowl management and conservation planning is dependent on accurate estimates of the spatio-temporal distribution of waterfowl during all stages of their life-cycles [22, 23]. Without such knowledge, it becomes nearly impossible to ensure that specific demands for resources (e.g., energy, necessary nutrients, roosting habitat) by migrating waterfowl can be met at appropriate points in time and space. This is of particular importance as mismatches between the need for resources and availability of those resources can have detrimental effects on survival and reproduction [22, 24–27]. Moreover, detailed knowledge of changes in migration phenology is necessary for guiding waterfowl harvest policies, particularly setting timing for seasons such that they correspond with occurrence of waterfowl. Therefore, it is important to understand how migration phenologies have changed in the past and how they may change in the future.

Waterfowl management in North America is administered within 4 major flyways: the Atlantic, Mississippi, Central, and Pacific flyways. Our study focused on the mid- to lower portion of the Central Flyway and encompassed Texas, Oklahoma, Kansas, Nebraska, and the eastern portion of New Mexico (east of the continental divide). Within this region, the U.S. Fish and Wildlife Service manages numerous conservation units (hereafter, refuges) many of which were established or are managed primarily for waterfowl conservation [28, 29]. As global warming continues, information on waterfowl migration phenologies within this flyway and how they may relate to changes in the climate will be critical for guiding future waterfowl harvest policies, management, and conservation strategies throughout central North America. As a significant number of the refuges within this region either currently monitor their waterfowl numbers or have done so in the past, they provide an opportunity to examine long-term changes in waterfowl migration phenology in this flyway. In this study, we utilized historical (1955–2008) nonbreeding waterfowl survey data from the Central Flyway to produce the first comprehensive summary of phenological changes in spring and autumn waterfowl migration for this region. Changes in spring and autumn migration phenology were evaluated with respect to time and site-specific temperatures.

## Methods

### Data collection and processing

We gathered all available nonbreeding waterfowl survey data from 21 refuges providing at least 5 years of data within the mid- to lower portion of the Central Flyway (see [30] for detailed descriptions of data acquisition and entry). Survey data consisted of aerial and ground counts conducted during 1955–2008; data spanned from 7 to 54 years (mean ± SD: 26.6 ± 12.7) with the number of years with data ranging from 7 to 47 (mean ± SD: 24.0 ± 9.4) among sites. Only sites at mid- to lower latitudes were included because refuges at higher latitudes harbor many breeding waterfowl that makes it difficult to identify migrating waterfowl. Because few refuges conducted regular surveys during September or April, we restricted data to October–December for the autumn-winter (hereafter, autumn) migration period and January–March for the winter-spring (hereafter, spring) migration period to obtain comparable data for as many refuges as possible, while still including most of the autumn and spring migrations for the majority of waterfowl species of the Central Flyway [31]. Further details on the waterfowl survey data can be found in [30, 32].

We included all waterfowl species for which data were available in our analyses. However, due to the relatively recent split of the Canada goose complex into 2 different species [33], very few surveys in our study distinguished between Canada Geese (*Branta canadensis*) and Cackling Geese (*B*. *hutchinsii*). We therefore combined these 2 species into 1 group (Canada geese) for all analyses. Similarly, only 1 refuge differentiated between Snow Geese (*Anser caerulescens*) and Ross’s Geese (*A*. *rossii*); therefore, we analyzed them separately for this refuge, but treated them as a single group (light geese) for all other refuges. We also combined Greater and Lesser Scaup (*Aythya marila* and *A*. *affinis*; hereafter, scaup) and Common and Barrow’s Goldeneyes (*Bucephala clangula* and *B*. *islandica*; hereafter, goldeneyes) because they are closely related and difficult to visually separate in the field. All group sums (Canada geese, light geese, scaup, and goldeneyes) were calculated on raw count data for each individual survey before any other calculations were performed. We propagated missing values through summations so that any sum based upon a missing count value resulted in a missing value.

All individual surveys that were found to be incomplete (i.e., when only a portion of the usual survey area was surveyed) were excluded from all analyses. Following [32], we considered counts for which the proportion of unidentified birds was indeterminable or exceeded 10% as too unreliable and therefore excluded those data from all analyses. A few sites experienced minor increases in survey area over time (see S1–S4 Tables), and if not correctable by subtracting the counts for the added area, we used uncorrected counts. However, in no case was there any discernible effect on the relative number of counted individuals of any species among months as a result of increased survey area (Kent Andersson, Oklahoma State University, unpublished data). The frequency of waterfowl surveys varied within and among sites, from weekly to monthly surveys. Eleven sites offered only monthly surveys and only 3 sites consistently offered weekly surveys for greater than 50% of their time series. The timing of surveys within months often varied within sites as well. Therefore, we calculated monthly averages to make data consistent among and within sites.

To assess changes in migration phenology over time, we used changes in the timing of peak abundance, as indexed by the monthly count averages, as an indicator of population-level changes in migration timing. We defined peak abundance month as the month with the greatest count average within each refuge, species, and year for spring and autumn migration periods separately (hereafter, spring peak and autumn peak, respectively). Thus, for each specific refuge, species, year, and migration season, the month with the greatest count average within the 3-month period was considered the peak abundance month. Any individual spring or autumn migration period for any refuge, species, and year that did not provide count averages for all 3 months or for which a single peak month could not be identified (i.e., the greatest count average was represented in more than 1 month) was excluded from all analyses.

Peak abundance month exhibits several traits that makes it suitable for analyzing changes in migration phenology at the population level: 1) it represents when a large fraction of the population that uses a particular site can be found there and therefore, likely reflects high resource demands at that site; 2) it is robust to changes in survey effort and population size, which date of first arrival or last departure are not [5, 34–36]; and 3) unlike the mean or median passage date, peak abundance month is also robust against truncated distributions, such as the current survey data, as long as the distribution of monthly averages is unimodal or the highest peak occurs within the defined spring or autumn migration periods. In our case, this would likely be true for most refuges and species. Peaks occurring outside the defined migration periods could potentially present a problem. However, because we treated peak month as an ordered categorical variable, as long as the highest peak within the defined migration period occurs in the month closest to the true peak outside the defined period, the validity and interpretation of the results remain unchanged. Cases like these merely change the interpretation of the first or last months (i.e., categories) to mean that the peak occurred in or before the first month or in or after the last month, respectively, rather than just within the month itself. Further, an evaluation of all the refuges in our study offering any survey data outside October–March, revealed bimodality with the highest peak outside the defined periods were rare, and cases where the internal peak did not fall at the relevant boundary occurred in only 3% of cases (see S1 Text for further details). For specific species where particular refuges serve as wintering sites, spring and autumn peaks naturally represent peaks in the number of wintering birds rather than actively migrating birds. However, as the implications are the same from the perspective of migration phenology (i.e., an earlier peak indicates advanced arrival of migrating birds and a later peak indicates a delayed arrival), we did not distinguish between them here.

**Fig 1:**
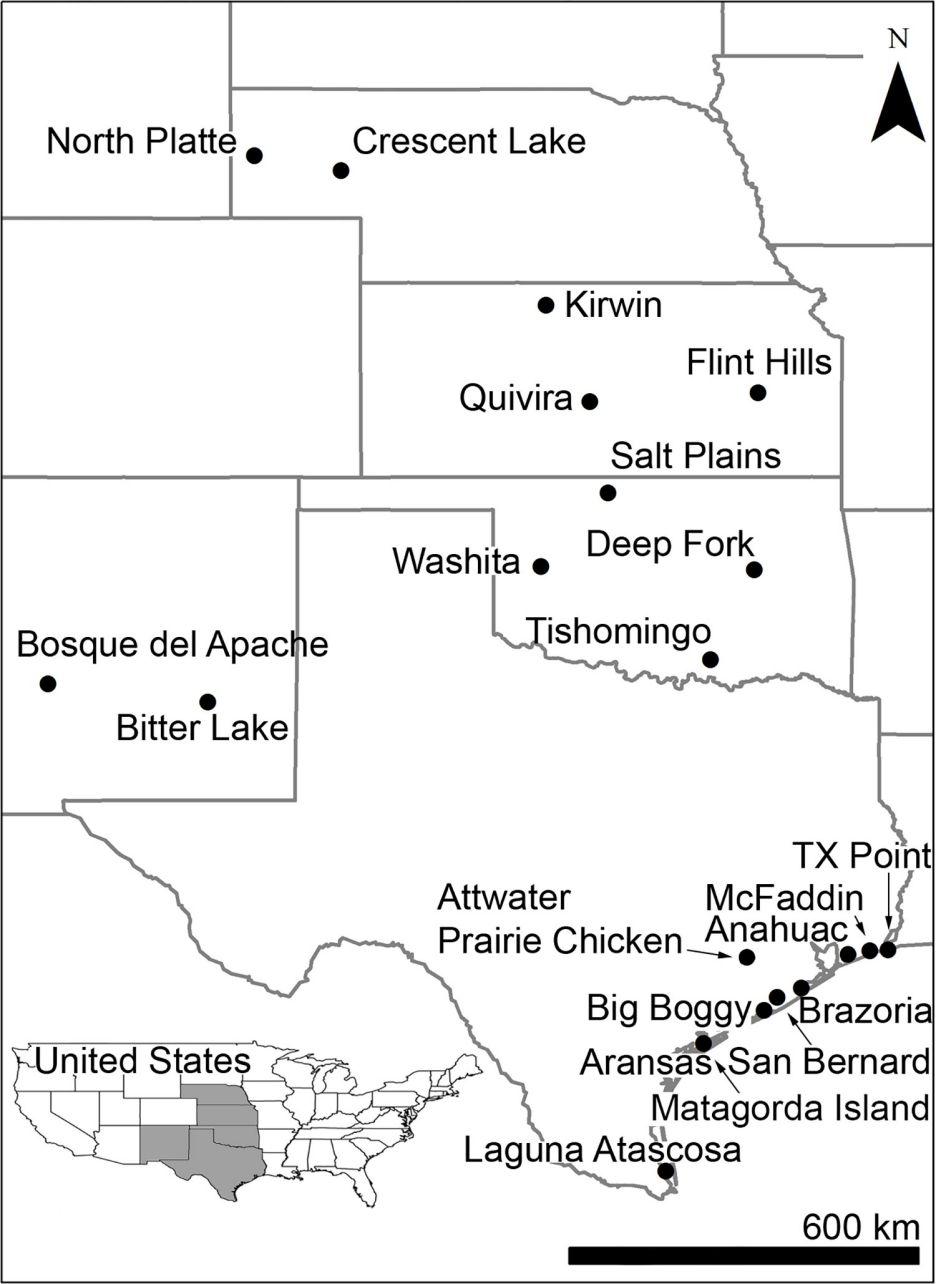
Locations of the National Wildlife Refuges included in this study. Abbreviation represents Texas Point (TX Point).

The more sporadically a specific waterfowl species occurs at a given location and the lower the average peak abundance, the more likely it is that random events will dominate any observed patterns in peak abundance, obscuring actual changes in migration phenology. Therefore, we limited phenology analyses to species-refuge combinations with a total of at least 5 non-zero data points, less than 25% zeros, and an average peak count of 200 birds or more for the migration season considered. Of the 21 sites, 2 were located in Nebraska (North Platte and Crescent Lake), 3 in Kansas (Kirwin, Flint Hills, and Quivira), 4 in Oklahoma (Salt Plains, Washita, Deep Fork, and Tishomingo), 2 in New Mexico (Bosque del Apache and Bitter Lake), and 10 in Texas (Texas Point, Attwater Prairie Chicken, McFaddin, Anahuac, Brazoria, San Bernard, Big Boggy, Matagorda Island, Aransas, and Laguna Atascosa; Fig 1). Although Matagorda Island is technically a unit of Aransas, it was surveyed independently and therefore, we considered it separately. All 21 sites were designated as National Wildlife Refuges by the U.S. Fish and Wildlife Service.

By focusing on peak abundance month (as determined by the greatest monthly count average), the only assumption needed for our analyses was that surveys were methodologically and spatially consistent within each distinct 3-month period. Most of the included data sets show a relatively high degree of methodological and spatial consistency over their entire data series [32]. This consistency becomes even greater when differences between years and migration seasons are not considered (Kent Andersson, Oklahoma State University, unpublished data). Moreover, even the absence of a written survey protocol (a fairly common problem among these surveys; [32]) is unlikely to lead to such vast differences in methodology over as short a time interval as 3 consecutive months as to alter the rank order of the months. Hence, we were confident that the identified peak month corresponded to the month with the greatest average number of waterfowl for each species and location.

To assess the influence of site-specific temperature on waterfowl migration phenology, we downloaded daily maximum temperatures for each refuge centroid from the PRISM (Parameter-elevation Regressions on Independent Slopes Model) [37] data set for the spring (January–March) and autumn (October–December) migration periods for all relevant years (PRISM Climate Group, Oregon State University, http://prism.oregonstate.edu, created 19 April 2021). Because data prior to 1981 were not available from the PRISM data set, we downloaded daily maximum temperatures for the nearest weather station with available data for years prior to 1981 from the National Oceanic and Atmospheric Administration’s Global Historical Climatology Network—Daily Database [38, 39] (https://www.ncdc.noaa.gov/cdo-web/, accessed 19 April 2021). To ensure these temperature data were comparable to PRISM data, we also downloaded overlapping temperature data for 1981–2008 and performed linear regressions on monthly averages for the overlapping data; and because all slopes were near 1.0 (range: 0.98–1.01), we considered these data sources comparable. We then calculated the average daily maximum temperature for each migration season, year, and refuge. Thus, each resulting temperature average corresponded to an entire spring or autumn migration season for a specific year and refuge.

### Data analyses

We analyzed each refuge and species for changes in spring (*n* = 187) and autumn (*n* = 194) peak by year and average daily maximum temperature separately using PROC LOGISTIC in SAS 9.4 (SAS Institute, Inc., Cary, NC). Data were either binomial (i.e., when all peaks fell within 2 months; spring: *n* = 67, autumn: *n* = 69) or trinomial (i.e., when all 3 months were represented; spring: *n* = 100, autumn: *n* = 114). For the purpose of statistical analysis, the cases where all peaks fell within the same month were trivial, as no temporal change in peak could be detected in those cases (spring: *n* = 20, autumn: *n* = 11).

Binomial data sets were analyzed using logistic regression with penalized maximum likelihood estimation. The main reason for using penalized maximum likelihood was to obtain finite maximum likelihood estimates for data sets with complete separation [40] (*n* = 4 and *n* = 2 with year and average maximum daily temperature as explanatory variables, respectively, for both spring and autumn), but it has the added benefit of reducing bias resulting from small sample size [41]. Trinomial data sets were analyzed using ordinal logistic regression with a proportional odds version of the cumulative logit model [42]. This was based on the assumption of an underlying latent continuous response variable (i.e., timing of abundance peak) that satisfied an ordinary regression model with a similar dispersion across the continuum of the predictor variables (i.e., year or temperature) [43–45]. Such cumulative logit models are sensitive to location effects rather than changes in variability with changing values of the predictor variable [42]. Thus, this assumption seemed reasonable for our data where the underlying latent variable, timing of abundance peak, undoubtedly was continuous and unlikely to differ dramatically in dispersion over relevant years or temperatures. Moreover, a score test of the proportional odds assumption (available in PROC LOGISTIC [46]) performs poorly when data are sparse, such as when comparatively few observations fall in 1 of the response categories or in the case of continuous predictors [45]. As our predictor variables were continuous and data were often sparse in 1 response category, the validity of any score test for the assumption of proportional odds would be highly questionable. Therefore, we assumed the proportional odds assumption to be valid for all trinomial data sets *a priori*.

Penalized maximum likelihood estimation for ordinal logistic regression was not supported in SAS 9.4 [46]. We therefore transformed the single trinomial data set that exhibited complete separation (i.e., spring peaks by year for Ross’s Goose at Bitter Lake) to a binary data set by combining the single March peak with the 2 February peaks into 1 category before analysis. Because the underlying data exhibited a monotonic increase in peak month over time, this transformation was conservative. For ordinal categorical data, regression parameters often have asymmetric distributions when most observations fall in the highest or lowest category, when sample size is small, or when complete separation exists [40, 42]. Because many of our data sets exhibited 1 or more of these characteristics, we used likelihood ratio tests to identify statistically significant trends and based all confidence intervals on profile likelihood estimates [40, 42]. All modeled probabilities were cumulated over higher values of peak month; thus, a positive slope indicates that abundance peaks later in the season over time or with increasing temperature and a negative slope that it peaks earlier.

The power of a statistical test is dependent on the sample size with a smaller sample size resulting in lower power. Further, in ordinal logistic regression, fewer possible outcome categories also translate into lower power [42]. As our sample sizes were generally small and the number of outcome categories for the ordinal logistic regressions merely 3, it was likely that smaller shifts in migration phenology would not manifest as statistically significant. Hence, to identify general patterns for either year or temperature that may otherwise go undetected, we followed [1] and performed exact binomial tests on the distribution of all positive and negative slope values derived from the logistic regressions, regardless of whether statistical significance was obtained during the regression. We did this for regressions with year and temperature separately. Our null hypothesis was equal probability (i.e., *P* = 0.5) of a non-zero slope being positive or negative (only non-zero slopes were included in these analyses as slope values of zero were neither positive nor negative [1, 47]). For each explanatory variable (year and temperature), we performed this analysis on the entire data set, as well as on individual refuges across all species and on individual species across all refuges for spring and autumn migration periods, separately.

To investigate if the prevalence of advancements or delays in peak month by year or temperature was related to latitude, we used linear regression of the proportion of non-zero slopes (obtained from the logistic regressions) that indicated advancement (i.e., negative slopes) or delays (i.e., positive slopes) for each refuge and predictor variable as a function of refuge centroid latitude. In all cases, scatterplots of standardized predicted values vs. standardized residuals for the linear regressions indicated that these data met the assumptions of homogeneity of variance and linearity and the residuals were approximately normally distributed. The significance level was set to α = 0.05. All tests were two-tailed.

## Results

### Shifts over time

**Fig 2:**
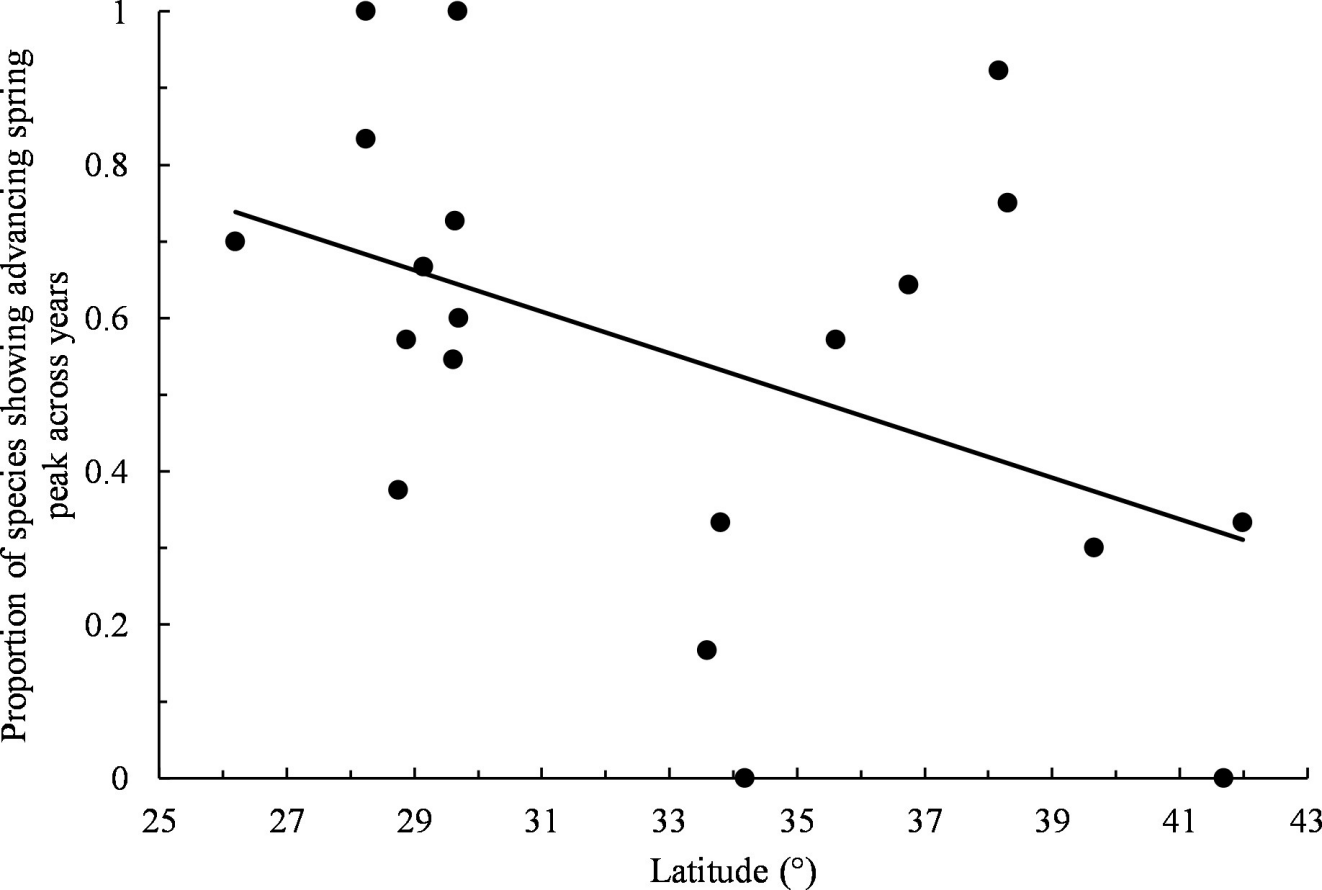
Proportion of species showing advancing spring peak across years by refuge latitude. Only non-zero slopes were included. Trend line obtained by least squares linear regression.

Of 187 spring data sets, 13% exhibited statistically significant changes in the timing of peak migration across years. The dominant pattern of change during spring was towards earlier migration peaks over time, both across all non-zero spring slopes (61% negative slopes, *n* = 167, exact binomial test: *P* = 0.005; S1 Table) and among statistically significant trends (80% negative slopes, *n* = 25). When examining non-zero slopes for individual species separately across all refuges, only Green-winged Teal (*Anas crecca*) displayed a dominance of slopes that was statistically significant with 78% of slopes indicating earlier spring peaks over time (Table 1). For individual refuges separately across all species, Aransas, Attwater Prairie Chicken, Matagorda Island, and Quivira displayed significantly skewed distributions of positive and negative trends, all of which indicated a dominance of shifts towards earlier spring peaks over time (83%, 100%, 100%, and 92% negative trends, respectively; Table 1). Species trends showing shifts towards earlier spring peaks over time became proportionally less common with increasing refuge latitude (least squares linear regression: *b* = -0.027, *r*^*2*^ = 0.20, *F*_*1*,*18*_ = 4.58, *P* = 0.046; Fig 2).

**Table 1:** Skewness in the distribution of slopes indicating advancements or delays in spring peak across years by waterfowl species and refuge.

Species	*n* ^a^	% neg^b^	*P* ^c^	Refuge	*n* ^a^	% neg ^b^	*P* ^c^
American Wigeon	14	43	0.79	Anahuac	11	55	1.00
Blue-winged Teal	11	82	0.07	Aransas	12	83	0.04
Bufflehead	1	100	1.00	Attwater Prairie Chicken	7	100	0.02
Canada geese	12	42	0.77	Big Boggy	8	38	0.73
Canvasback	4	75	0.63	Bitter Lake	6	17	0.22
Cinnamon Teal	1	0	1.00	Bosque del Apache	9	33	0.51
Common Merganser	5	40	1.00	Brazoria	9	67	0.51
Gadwall	13	77	0.09	Crescent Lake	1	0	1.00
Goldeneyes	1	0	1.00	Flint Hills	8	75	0.29
Greater White-fronted Goose	9	67	0.51	Kirwin	10	30	0.34
Green-winged Teal	18	78	0.03	Laguna Atascosa	10	70	0.34
Light geese	14	71	0.18	Matagorda Island	8	100	0.008
Mallard	10	40	0.75	McFaddin	11	73	0.23
Northern Pintail	16	50	1.00	North Platte	6	33	0.69
Northern Shoveler	14	71	0.18	Quivira	13	92	0.003
Redhead	8	88	0.07	Salt Plains	14	64	0.42
Ring-necked Duck	1	0	1.00	San Bernard	7	57	1.00
Ross’s Goose	1	0	1.00	Texas Point	5	60	1.00
Ruddy Duck	3	67	1.00	Tishomingo	5	0	0.06
Scaup	10	50	1.00	Washita	7	57	1.00
Snow Goose	1	0	1.00				

^a^ Only non-zero slopes were included as slope values of zero were neither positive nor negative [1, 47].

^b^ Percent non-zero logistic regression slopes that indicated advancement of spring abundance peak across years (i.e., negative slopes).

^c^ *P*-value from exact binomial test under the null hypothesis of equal probability (i.e., *P* = 0.5) of a non-zero slope being positive or negative. Tests were performed for each waterfowl species across all refuges and for each refuge across all species.

**Fig 3:**
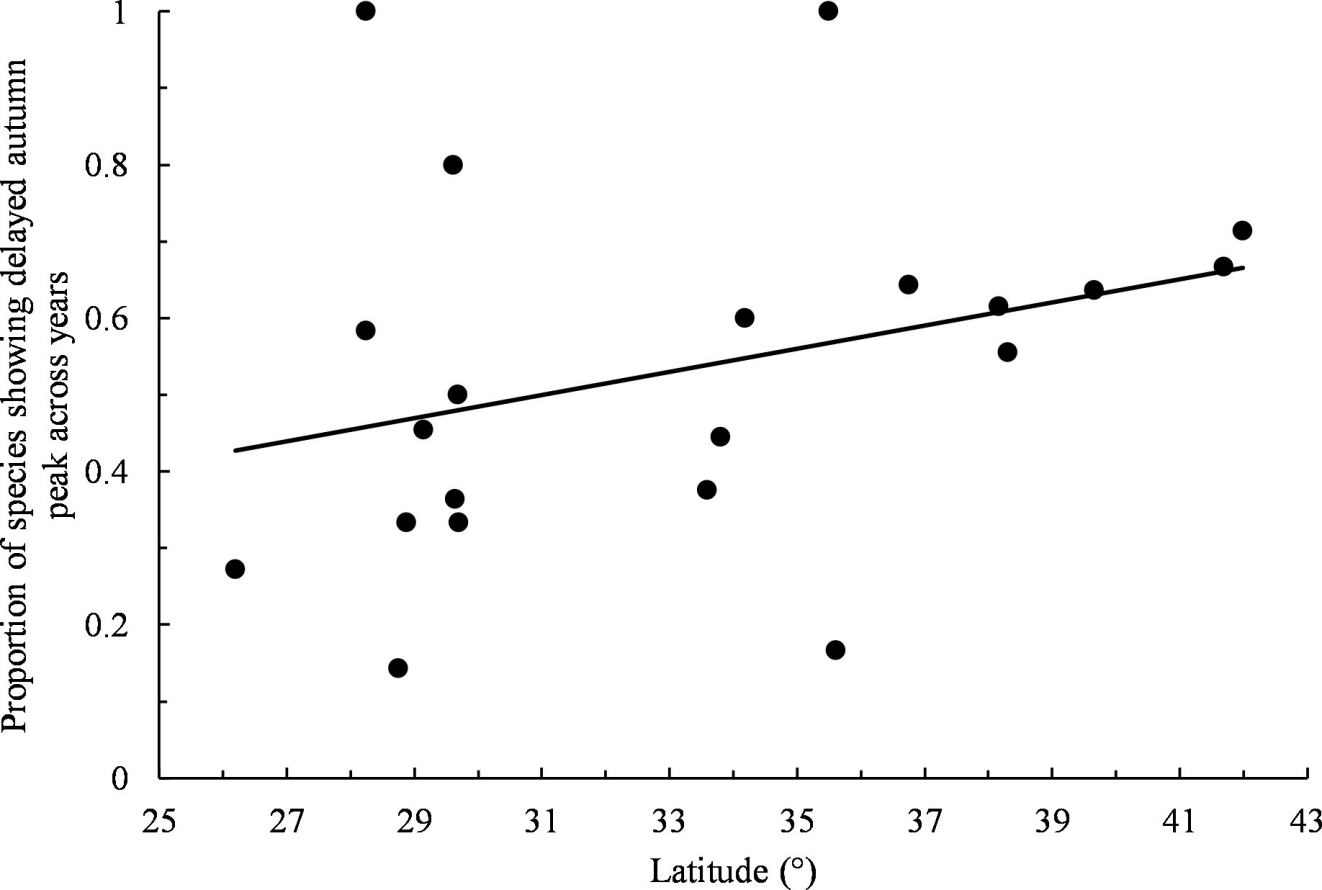
Proportion of species showing delayed autumn peak across years by refuge latitude. Only non-zero slopes were included. Trend line obtained by least squares linear regression.

For autumn, 9% of data sets (*n* = 194) exhibited statistically significant changes in the timing of peak migration across years, with 67% of significant trends (*n* = 18) indicating peaks occurring later over time. There was no obvious pattern in peak shifts when all non-zero slopes were considered (52% positive trends, *n* = 183, exact binomial test: *P* = 0.55; S2 Table). No individual species exhibited a statistically significant skew in its autumn distributions of positive and negative trends across all refuges (Table 2). When considering individual refuges separately across all species, only Matagorda Island showed a significantly skewed distribution of positive and negative trends with 100% of slopes indicating autumn peaks occurring later over time (Table 2). Species trends showing advancing autumn peaks over time showed a tendency to become proportionally less common with increasing refuge latitude, but the relationship was not statistically significant (least squares linear regression: *b* = 0.015, *r*^*2*^ = 0.10, *F*_*1*,*19*_ = 2.04, *P* = 0.17; Fig 3).

**Table 2:** Skewness in the distribution of slopes indicating advancements or delays in autumn peak across years by waterfowl species and refuge.

Species	*n* ^a^	% pos ^b^	*P* ^c^	Refuge	*n* ^a^	% pos ^b^	*P* ^c^
American Wigeon	18	50	1.00	Anahuac	10	80	0.11
Blue-winged Teal	12	50	1.00	Aransas	12	58	0.77
Bufflehead	1	0	1.00	Attwater Prairie Chicken	6	50	1.00
Canada geese	10	40	0.75	Big Boggy	7	14	0.13
Canvasback	4	25	0.63	Bitter Lake	8	38	0.73
Cinnamon Teal	1	0	1.00	Bosque del Apache	9	44	1.00
Common Merganser	3	33	1.00	Brazoria	11	45	1.00
Gadwall	16	44	0.80	Crescent Lake	3	67	1.00
Goldeneyes	3	33	1.00	Deep Fork	2	100	0.50
Greater White-fronted Goose	12	75	0.15	Flint Hills	9	56	1.00
Green-winged Teal	17	35	0.33	Kirwin	11	64	0.55
Light geese	13	69	0.27	Laguna Atascosa	11	27	0.23
Mallard	12	58	0.77	Matagorda Island	8	100	0.008
Northern Pintail	16	56	0.80	McFaddin	11	36	0.55
Northern Shoveler	17	47	1.00	North Platte	7	71	0.45
Redhead	10	80	0.11	Quivira	13	62	0.58
Ring-necked Duck	3	67	1.00	Salt Plains	14	64	0.42
Ross’s Goose	1	0	1.00	San Bernard	9	33	0.51
Ruddy Duck	3	67	1.00	Texas Point	6	33	0.69
Scaup	9	56	1.00	Tishomingo	10	60	0.75
Snow Goose	1	100	1.00	Washita	6	17	0.22
Wood Duck	1	100	1.00				

^a^ Only non-zero slopes were included as slope values of zero were neither positive nor negative [1, 47].

^b^ Percent non-zero logistic regression slopes that indicated delayed autumn abundance peak across years (i.e., positive slopes).

^c^ *P*-value from exact binomial test under the null hypothesis of equal probability (i.e., *P* = 0.5) of a non-zero slope being positive or negative. Tests were performed for each waterfowl species across all refuges and for each refuge across all species.

### Shifts in relation to temperature

**Fig 4:**
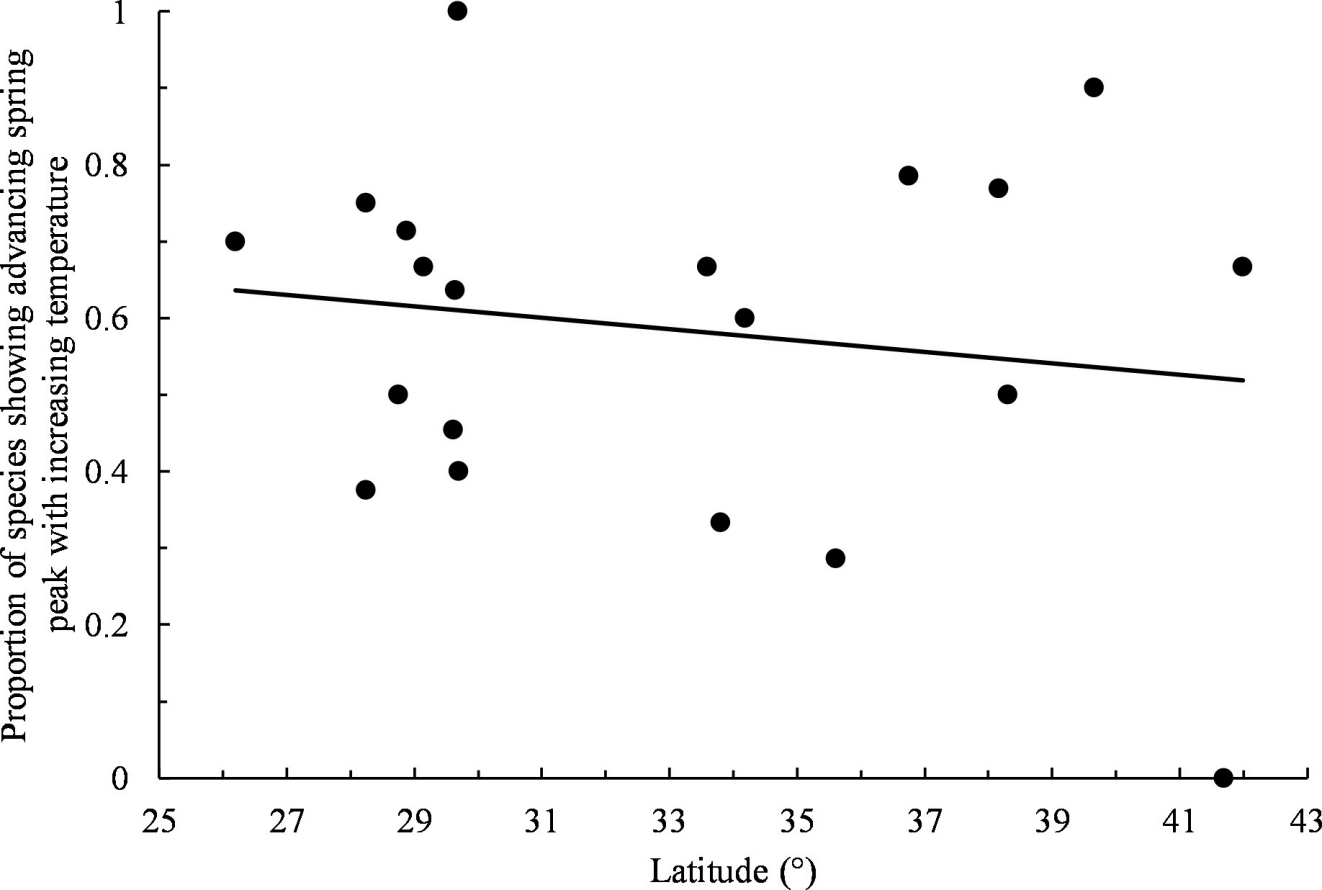
Proportion of species showing advancing spring peak with increasing temperature by refuge latitude. Only non-zero slopes were included. Trend line obtained by least squares linear regression.

The dominant pattern during spring was for higher temperatures being associated with earlier migration peaks, both across all spring data sets (63% negative slopes, *n* = 167, exact binomial test: *P* = 0.001; S3 Table) and among statistically significant trends (88% negative slopes, *n* = 8). However, only 4% of spring data sets (*n* = 187) exhibited statistically significant changes in the timing of peak migration with increasing temperatures. When examining positive and negative trends for individual species separately across all refuges, only Northern Pintail (*Anas acuta*) displayed a dominance of slopes that was statistically significant with 81% of slopes indicating earlier spring peaks with increasing temperatures (Table 3). For individual refuges across all species, Attwater Prairie Chicken and Kirwin displayed significantly skewed trend distributions of positive and negative slopes, with both showing earlier spring peaks with increasing temperatures as the dominant pattern (100% and 90% negative slopes, respectively; Table 3). Refuge latitude showed no discernable relationship with the proportion of species trends showing advancement in spring peak with increasing temperature (least squares linear regression: *b* = -0.007, *r*^*2*^ = 0.03, *F*_*1*,*18*_ = 0.46, *P* = 0.5; Fig 4).

**Table 3:** Skewness in the distribution of slopes indicating advancements or delays in spring peak with increasing temperature by waterfowl species and refuge.

Species	*n* ^a^	% neg ^b^	*P* ^c^	Refuge	*n* ^a^	% neg ^b^	*P* ^c^
American Wigeon	14	57	0.79	Anahuac	11	45	1.00
Blue-winged Teal	11	45	1.00	Aransas	12	75	0.15
Bufflehead	1	100	1.00	Attwater Prairie Chicken	7	100	0.02
Canada geese	12	58	0.77	Big Boggy	8	50	1.00
Canvasback	4	50	1.00	Bitter Lake	6	67	0.69
Cinnamon Teal	1	0	1.00	Bosque del Apache	9	33	0.51
Common Merganser	5	100	0.06	Brazoria	9	67	0.51
Gadwall	13	62	0.58	Crescent Lake	1	0	1.00
Goldeneyes	1	100	1.00	Flint Hills	8	50	1.00
Greater White-fronted Goose	9	56	1.00	Kirwin	10	90	0.02
Green-winged Teal	18	72	0.10	Laguna Atascosa	10	70	0.34
Light geese	14	71	0.18	Matagorda Island	8	38	0.73
Mallard	10	40	0.75	McFaddin	11	64	0.55
Northern Pintail	16	81	0.02	North Platte	6	67	0.69
Northern Shoveler	14	64	0.42	Quivira	13	77	0.09
Redhead	8	63	0.73	Salt Plains	14	79	0.06
Ring-necked Duck	1	0	1.00	San Bernard	7	71	0.45
Ross’s Goose	1	100	1.00	Texas Point	5	40	1.00
Ruddy Duck	3	100	0.25	Tishomingo	5	60	1.00
Scaup	10	50	1.00	Washita	7	29	0.45
Snow Goose	1	0	1.00				

^a^ Only non-zero slopes were included as slope values of zero were neither positive nor negative [1, 47].

^b^ Percent non-zero logistic regression slopes that indicated advancement of spring abundance peak with increasing site-specific average Jan–Mar daily maximum temperature (i.e., negative slopes).

^c^ *P*-value from exact binomial test under the null hypothesis of equal probability (i.e., *P* = 0.5) of a non-zero slope being positive or negative. Tests were performed for each waterfowl species across all refuges and for each refuge across all species.

**Fig 5:**
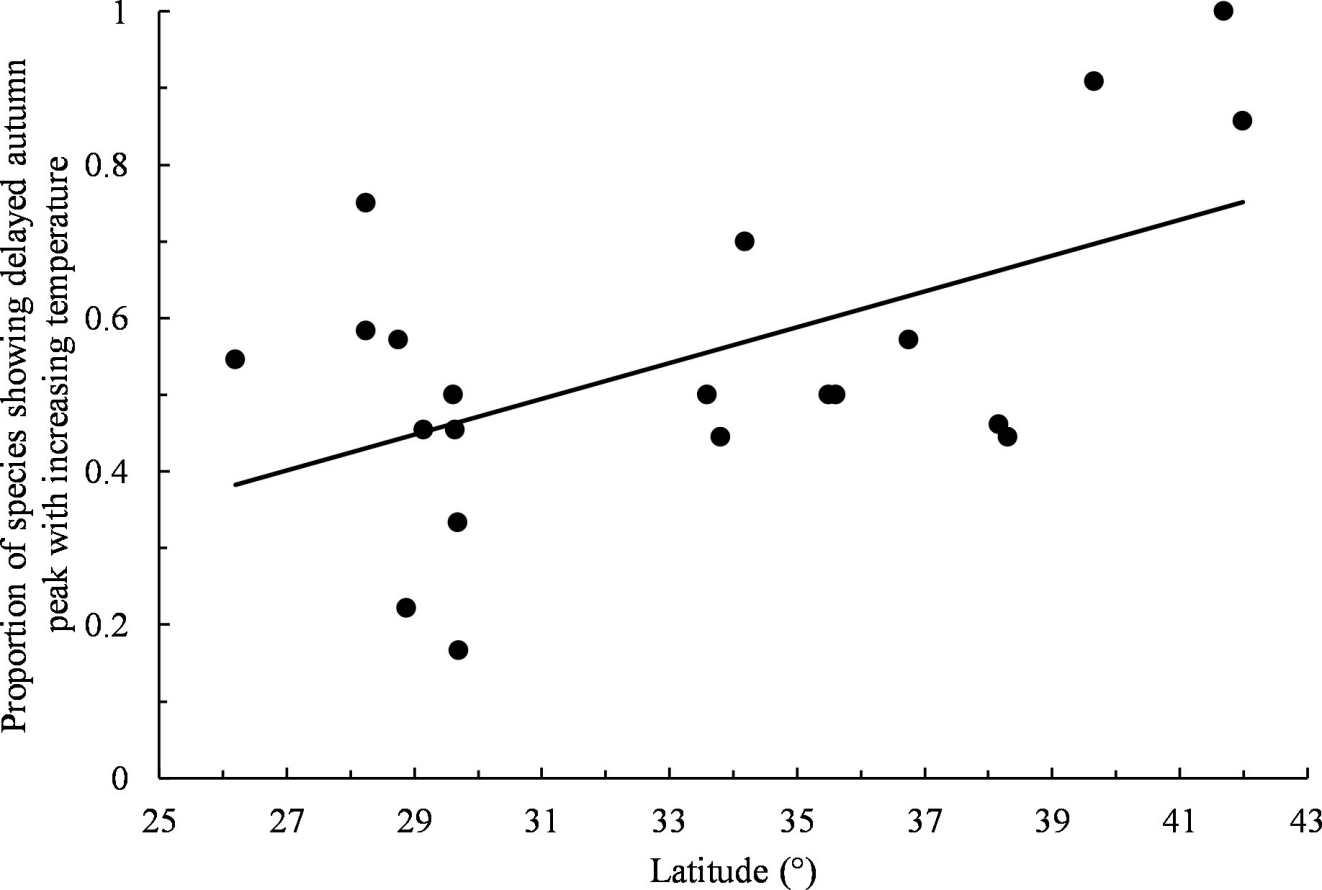
Proportion of species showing delayed autumn peak with increasing temperature by refuge latitude. Only non-zero slopes were included. Trend line obtained by least squares linear regression.

For autumn, 9% of data sets (*n* = 194), exhibited statistically significant changes in the timing of peak migration with increasing temperature, with 67% of significant trends (*n* = 18) indicating peaks occurring later with increasing temperature. There was no obvious pattern in peak shifts when all non-zero slopes were considered (54% positive trends, *n* = 183, exact binomial test: *P* = 0.30; S4 Table). No species exhibited a statistically significant skew in its autumn distributions of positive and negative trends across all refuges (Table 4). When considering individual refuges separately across all species, only Kirwin showed a significantly skewed distribution of positive and negative trends with 91% of slopes indicating autumn peaks occurring later with increasing temperature (Table 4). Species trends indicating delayed autumn peaks with increasing temperature became proportionally more common with increasing refuge latitude (least squares linear regression: *b* = 0.023, *r*^*2*^ = 0.30, *F*_*1*,*19*_ = 8.20, *P* = 0.010; Fig 5).

**Table 4:** Skewness in the distribution of slopes indicating advancements or delays in autumn peak with increasing temperature by waterfowl species and refuge.

Species	*n* ^a^	% pos ^b^	*P* ^c^	Refuge	*n* ^a^	% pos ^b^	*P* ^c^
American Wigeon	18	39	0.48	Anahuac	10	50	1.00
Blue-winged Teal	12	67	0.39	Aransas	12	58	0.77
Bufflehead	1	100	1.00	Attwater Prairie Chicken	6	33	0.69
Canada geese	10	60	0.75	Big Boggy	7	57	1.00
Canvasback	4	25	0.63	Bitter Lake	8	50	1.00
Cinnamon Teal	1	0	1.00	Bosque del Apache	9	44	1.00
Common Merganser	3	100	0.25	Brazoria	11	45	1.00
Gadwall	16	44	0.80	Crescent Lake	3	100	0.25
Goldeneyes	3	67	1.00	Deep Fork	2	50	1.00
Greater White-fronted Goose	12	58	0.77	Flint Hills	9	44	1.00
Green-winged Teal	17	65	0.33	Kirwin	11	91	0.01
Light geese	13	62	0.58	Laguna Atascosa	11	55	1.00
Mallard	12	75	0.15	Matagorda Island	8	75	0.29
Northern Pintail	16	50	1.00	McFaddin	11	45	1.00
Northern Shoveler	17	29	0.14	North Platte	7	86	0.13
Redhead	10	60	0.75	Quivira	13	46	1.00
Ring-necked Duck	3	33	1.00	Salt Plains	14	57	0.79
Ross’s Goose	1	0	1.00	San Bernard	9	22	0.18
Ruddy Duck	3	67	1.00	Texas Point	6	17	0.22
Scaup	9	67	0.51	Tishomingo	10	70	0.34
Snow Goose	1	100	1.00	Washita	6	50	1.00
Wood Duck	1	0	1.00				

^a^ Only non-zero slopes were included as slope values of zero were neither positive nor negative [1, 47].

^b^ Percent non-zero logistic regression slopes that indicated delayed autumn abundance peak with increasing site-specific average Oct–Dec daily maximum temperature (i.e., positive slopes).

^c^ *P*-value from exact binomial test under the null hypothesis of equal probability (i.e., *P* = 0.5) of a non-zero slope being positive or negative. Tests were performed for each waterfowl species across all refuges and for each refuge across all species.

## Discussion

Given that Earth’s climate is projected to continue changing as a result of ongoing global warming [48], it is critical that we identify and understand changes in migration phenologies of migratory birds. For migratory waterfowl, such information is particularly important because stopover habitats used during migration are critical for acquisition of nutrients necessary for continuing migration, successful reproduction, and survival [22, 24–27]. Changing migration phenologies may generate mismatches in their ability to rely on such habitats. Our results indicated that the timing of waterfowl migration in the mid- to lower portion of the Central Flyway has undergone significant changes since the 1950s. Statistically significant trends for changes in peak abundance month were relatively rare during both autumn and spring periods (4–13% of all data sets). However, the dominant pattern for changes in spring migration was that of peak abundance month occurring earlier over time and with increasing temperatures, both for statistically significant trends only and across all non-zero slopes. During autumn migration, only statistically significant trends showed any discernable pattern, with delays in peak abundance month being more common than advancements over time and with increasing temperatures.

Given the coarseness of the temporal resolution of the dependent variable (i.e., peak abundance month) and the necessity to treat it as an ordinal categorical variable with only 3 possible outcomes for each migration period, it was not unexpected that relatively few species-refuge trends manifested as statistically significant. Even with our generally relatively long survey series (mean ± SD: 26.6 ± 12.7 years), only very strong relationships would likely result in statistically significant trends. Given this, the number of detected phenological changes was surprisingly high and suggests that rather dramatic phenological shifts in waterfowl migration have occurred in North America during the latter part of the twentieth century.

With global temperatures expected to continue increasing [48], we expect migration timing to continue changing for many waterfowl species, leading to gradual changes in the timing of their occurrence at specific refuges. Thus, for individual refuges, resource management may need to be adjusted such that availability of resources match waterfowl abundances and requirements. Such optimization of refuge management would maximize conservation as well as fiscal outcomes. Further, the timing of local harvest seasons must coincide with presence of waterfowl in great enough numbers to allow rewarding hunting opportunities or it may negatively affect hunter participation. This, in turn, may directly lead to reduced funding for waterfowl conservation through loss of revenue from sales of Federal Migratory Bird Hunting Conservation Stamps (i.e., duck stamps; [49]).

Numerous studies have documented advancement in spring migration in birds including some waterfowl [5–9]. Our results, indicating a widespread advancement in the timing of spring migration for waterfowl in the Central Flyway that appears connected to increasing temperatures, agree well with the general consensus of these previous studies. The apparent northward shifts in wintering distribution in several waterfowl species over the last century [31, 50] could explain at least some of this pattern as it may greatly accelerate the apparent migratory passage [8]. We also found that the proportion of trends indicating advancements versus delays in spring peak decreased with increasing latitude. This appears congruent with the findings of [8].

Mismatches between the need for and availability of resources required for successful breeding can have detrimental effects on a species reproductive success [51–53]. Hence, advancing spring waterfowl migration may be explained as an adaptive response if the conditions required for successful breeding, such as the availability of food and open water, occurred earlier over time. Indeed, the last century has seen increasing temperatures [48] concomitant with a gradual advancement of spring [54–56] and corresponding advancements of phenological events in many plants and animals [2, 3, 57–59] at a range of latitudes. However, as migrating waterfowl cannot have direct knowledge of the conditions at breeding areas, this also requires corresponding advancements in migration cues or needed resources along the migration route, or detrimental mismatches may still result [51].

The costs and benefits associated with and ecological and environmental constraints on autumn migration timing are poorly understood and likely highly variable depending on the species’ life history strategy [7, 10]. Generally, waterfowl migrate from breeding grounds to avoid deteriorating conditions linked to declining temperatures as winter approaches [60]. Indeed, temperature and snow cover as well as associated large-scale weather patterns have been identified as important factors affecting the timing of autumn migration for several waterfowl species [19, 61–64]. While temperature and related weather phenomena may be the ultimate drivers of autumn migration, many other factors, such as wind speed and direction [63, 65, 66], precipitation [66], human disturbance [67, 68], competition [69, 70], and predation pressure [71] could influence a specific species’ autumn migration phenology, leading to complex patterns [72].

Unfortunately, published data on changes in autumn migration phenology for waterfowl are scarce. What data exist suggest that there is much variation but delays appear more common than advancements [8, 13, 18, 19]. Our results confirmed this general pattern with significant delays being twice as common as advances, and with seasonal averages of local temperatures appearing to have some explanatory value. That delays in autumn peaks appeared more affected by temperature at higher latitudes support the theory that temperatures below freezing and snow cover are important drivers behind autumn migration for many waterfowl species in central North America. As the likelihood of lasting snow cover and extended periods of temperatures below freezing decreases with decreasing latitude [38, 39] (https://www.ncdc.noaa.gov/cdo-web/, accessed 16 July 2021), the effects of warming temperatures on the timing of autumn migration may be less pronounced at lower latitudes.

Given the variation in latitude and data series length and time span among refuges, it was not surprising that few species showed significantly skewed trend distributions across refuges. It was, however, noteworthy that 4 refuges exhibited significantly skewed trend distributions across species towards advancing spring peaks over time. This intimates that advancing spring migration may be widespread among species.

By necessity, studies of migration phenologies for larger birds like waterfowl generally rely on counts of actively migrating birds at stopover sites and therefore miss any birds that do not stop at the survey location, as well as individual birds that arrive and depart between consecutive surveys. This was the case for most waterfowl counts included in our analyses as well. But, it is generally assumed that counts of birds at stopover sites provide a good index of the actual number of birds that migrate past, although this remains an untested assumption [73].

Survey data from stopover or wintering sites may also involve different populations with different migration phenologies of the same species whose population sizes have changed asynchronously over time. This can cause apparent phenological shifts even if the respective migration phenologies remain unchanged over time. Unfortunately, reliable data for historical changes in different sub-populations of waterfowl species utilizing the Central Flyway appear rare to non-existent [31]. Given the large differences in biology of different waterfowl species, we judged this scenario to be an unlikely explanation of the generally consistent patterns across many species. Similarly, large-scale changes in relative dominance by groups divided by age, sex, or breeding investment (single versus multiple broods and successful versus unsuccessful nesting attempts) are unlikely to offer a cogent explanation of the observed patterns.

Our study represents the first large-scale analysis of changes in migration phenology for waterfowl in the Central Flyway. We show that waterfowl migrating within the Central Flyway have experienced significant changes in their spring and autumn migration phenology since the 1950s and that these changes appear linked to warming temperatures. As migration phenologies change, it is critical that management and conservation efforts are realigned to match the changing spatiotemporal distribution of waterfowl; otherwise, mismatches with critical food and habitat resources needed by migrating and wintering waterfowl may occur.

## Supporting information

S1 Table Phenological changes in spring abundance peak across years for waterfowl at Central Flyway National Wildlife Refuges. (DOCX)

S2 Table Phenological changes in autumn abundance peak across years for waterfowl at Central Flyway National Wildlife Refuges. (DOCX)

S3 Table Phenological changes in spring abundance peak with increasing temperature for waterfowl at Central Flyway National Wildlife Refuges. (DOCX)

S4 Table Phenological changes in autumn abundance peak with increasing temperature for waterfowl at Central Flyway National Wildlife Refuges. (DOCX)

S1 Text Further details on the choice of phenological parameter. (DOCX)

## References

[1] Parmesan C, Yohe G. A globally coherent fingerprint of climate change impacts across natural systems. Nature. 2003;421:37–42. doi: 10.1038/nature01286 12511946

[2] Root TL, Price JT, Hall KR, Schneider SH, Rosenzweig C, Pounds JA. Fingerprints of global warming on wild animals and plants. Nature. 2003;421:57–60. doi: 10.1038/nature01333 12511952

[3] Menzel A, Sparks TH, Estrella N, Koch E, Aasa A, Ahas R, et al. European phenological response to climate change matches the warming pattern. Glob Chang Biol. 2006;12:1969–1976.

[4] Parmesan C. Ecological and evolutionary responses to recent climate change. Annu Rev Ecol Evol Syst. 2006;37:637–669.

[5] Lehikoinen E, Sparks TH, Zalakevicius M. Arrival and departure dates. Adv Ecol Res. 2004;35:1–31.

[6] Rubolini D, Møller AP, Rainio K, Lehikoinen E. Intraspecific consistency and geographic variability in temporal trends of spring migration phenology among European bird species. Clim Res. 2007;35:135–146.

[7] Lehikoinen E, Sparks TH. Changes in migration. In: Møller AP, Fiedler W, Berthold P, editors. Effect of climate change on birds. London: Oxford University Press; 2010. pp. 89–112.

[8] Bitterlin LR, Van Buskirk J. Ecological and life history correlates of changes in avian migration timing in response to climate change. Clim Res. 2014;61:109–121.

[9] Usui T, Butchart SH, Phillimore AB. Temporal shifts and temperature sensitivity of avian spring migratory phenology, a phylogenetic meta-analysis. J Anim Ecol. 2017;86:250–261. doi: 10.1111/1365-2656.12612 27859281 PMC6849580

[10] Pearce-Higgins JW, Green RE. Birds and climate change: impacts and conservation responses. Cambridge (England): Cambridge University Press; 2014. doi: 10.1111/gcb.12559

[11] Gallinat AS, Primack RB, Wagner DL. Autumn, the neglected season in climate change research. Trends Ecol Evol. 2015;30:169–176. doi: 10.1016/j.tree.2015.01.004 25662784

[12] Cox GW. Bird migration and global change. Washington DC: Island Press; 2010.

[13] Lehikoinen A, Jaatinen K. Delayed autumn migration in Northern European waterfowl. J Ornithol. 2012;153:563–570.

[14] Guillemain M, Pöysä H, Fox AD, Arzel C, Dessborn L, Ekroos J, et al. Effects of climate change on European ducks: what do we know and what do we need to know? Wildlife Biol. 2013;19:404–419. doi: 10.2981/12-118

[15] Murphy-Klassen HM, Underwood TJ, Sealy SG, Czyrnyj AA. Long-term trends in spring arrival dates of migrant birds at Delta Marsh, Manitoba, in relation to climate change. Auk. 2005;122:1130–1148.

[16] Swanson DL, Palmer JS. Spring migration phenology of birds in the Northern Prairie region is correlated with local climate change. J Field Ornithol. 2009;80:351–363.

[17] DeLeon RL, DeLeon EE, Rising GR. Influence of climate change on avian migrants’ first arrival dates. Condor. 2011;113:915–923.

[18] Reese JG, Weterings R. Waterfowl migration chronologies in central Chesapeake Bay during 2002–2013. Wilson J Ornithol. 2018;130:52–69.

[19] Thurber BG, Roy C, Zimmerling JR. Long-term changes in the autumn migration phenology of dabbling ducks in southern Ontario and implications for waterfowl management. Wildlife Biol. 2020;wlb.00668. doi: 10.2981/wlb.00668

[20] Notaro M, Schummer M, Zhong Y, Vavrus S, Van Den Elsen L, Coluccy J, et al. Projected influences of changes in weather severity on autumn-winter distributions of dabbling ducks in the Mississippi and Atlantic flyways during the twenty-first century. PLoS ONE. 2016;11(12):e0167506. doi: 10.1371/journal.pone.0167506 27959911 PMC5154525

[21] Hurlbert AH, Liang Z. Spatiotemporal Variation in Avian Migration Phenology: Citizen Science Reveals Effects of Climate Change. PLoS ONE. 2012;7(2):e31662. doi: 10.1371/journal.pone.0031662 22384050 PMC3285173

[22] Kirby JS, Stattersfield AJ, Butchart SH, Evans MI, Grimmett RF, Jones VR, et al. Key conservation issues for migratory land- and waterbird species on the world’s major flyways. Bird Conserv Int. 2008;18:S49–S73.

[23] Williams CK, Dugger BD, Brasher MG, Coluccy JM, Cramer DM, Eadie JM, et al. Estimating habitat carrying capacity for migrating and wintering waterfowl: considerations, pitfalls and improvements. Wildfowl Special Issue 2014;4:407–435.

[24] Newton I. Population limitation in migrants. Ibis. 2004;146:197–226.

[25] Newton I. Can conditions experienced during migration limit the population levels of birds? J Ornithol. 2006;147:146–166.

[26] Arzel C, Elmberg J, Guillemain M. Ecology of spring-migrating Anatidae: a review. J Ornithol. 2006;147:167–184.

[27] Stafford JD, Janke AK, Anteau MJ, Pearse AT, Fox AD, Elmberg J, et al. Spring migration of waterfowl in the Northern Hemisphere: a conservation perspective. Wildfowl Special Issue. 2014;4:70–85.

[28] Linduska JP, editor. Waterfowl tomorrow. Washington DC: U.S Department of the Interior, Fish and Wildlife Service; 1964.

[29] Scott JM, Loveland T, Gergely K, Strittholt J, Staus N. National Wildlife Refuge System: ecological context and integrity. Nat Resour J. 2004;44:1041–1066.

[30] Andersson K, Davis CA, Harris G, Haukos DA. Nonbreeding duck use at Central Flyway National Wildlife Refuges. J Fish Wildl Manag. 2018;9:45–64.

[31] Baldassarre GA. Ducks, geese, and swans of North America. Baltimore (MD): Johns Hopkins University Press; 2014.

[32] Andersson K, Davis CA, Harris G, Haukos DA. An assessment of non-breeding waterfowl surveys on National Wildlife Refuges in the Central Flyway. Wildl Soc Bull. 2015;39:79–86.

[33] Banks RC, Cicero C, Dunn JL, Kratter AW, Rasmussen PC, Remsen JV, et al. Forty-fifth supplement to the American Ornithologists’ Union Checklist of North American Birds. Auk. 2004;121:985–995.

[34] Sparks TH, Roberts DR, Crick HQ. What is the value of first arrival dates of spring migrants in phenology? Avian Ecol Behav. 2001;7:75–85.

[35] Butler CJ. The disproportionate effect of global warming on the arrival dates of short‐distance migratory birds in North America. Ibis. 2003;145:484–495.

[36] Tryjanowski P, Kuźniak S, Sparks TH. What affects the magnitude of change in first arrival dates of migrant birds? J Ornithol. 2005;146:200–205.

[37] Daly C, Halbleib M, Smith JI, Gibson WP, Doggett MK, Taylor GH, et al. Physiographically-sensitive mapping of temperature and precipitation across the conterminous United States. Int J Climatol. 2008;28:2031–2064.

[38] Menne MJ, Durre I, Korzeniewski B, McNeal S, Thomas K, Yin X, et al. Global Historical Climatology Network—Daily (GHCN-Daily), Version 3 [Internet]. NOAA National Climatic Data Center; 2012 [cited 2021 Apr 19]. Available from: 10.7289/V5D21VHZ

[39] Menne MJ, Durre I, Vose RS, Gleason BE, Houston TG. An overview of the global historical climatology network-daily database. J Atmos Ocean Technol. 2012;29:897–910. doi: 10.1175/JTECH-D-11-00103.1

[40] Heinze G, Schemper M. A solution to the problem of separation in logistic regression. Stat Med. 2002;21:2409–2419. doi: 10.1002/sim.1047 12210625

[41] Firth D. Bias reduction of maximum likelihood estimates. Biometrika. 1993;80:27–38.

[42] Agresti A. Analysis of ordinal categorical data. 2nd ed. Hoboken (NJ): Wiley; 2010.

[43] McCullagh P. Regression models for ordinal data. J R Stat Soc Series B. 1980;42:109–142.

[44] Anderson JA, Philips PR. Regression, discrimination and measurement models for ordered categorical variables. Appl Stat. 1981;30:22–31.

[45] Peterson B, Harrell FE Jr. Partial proportional odds models for ordinal response variables. Appl Stat. 1990;39:205–217.

[46] SAS Institute Inc. SAS/STAT 14.3 user’s guide. Cary (NC): SAS Institute Inc.; 2017.

[47] Parmesan C, Ryrholm N, Stefanescu C, Hillket JK, et al. Poleward shifts in geographical ranges of butterfly species associated with regional warming. Nature 1999;399:579–583.

[48] IPCC. Climate Change 2013: The physical science basis. Contribution of Working Group I to the Fifth Assessment Report of the Intergovernmental Panel on Climate Change. Stocker TF, Qin D, Plattner GK, Tignor M, Allen SK, Boschung J, et al. editors. Cambridge (England): Cambridge University Press; 2013.

[49] Vrtiska MP, Gammonley JH, Naylor LW, Raedeke AH. Economic and conservation ramifications from the decline of waterfowl hunters. Wildl Soc Bull. 2013;37:380–388. doi: 10.1002/wsb.245

[50] Meehan TD, Kaminski RM, Lebaron GS, Michel NL, Bateman BL, Wilsey CB. Half-century winter duck abundance and temperature trends in the Mississippi and Atlantic Flyways. J Wildl Manage. 2021;85:713–722. doi: 10.1002/jwmg.22023

[51] Durant JM, Hjermann DØ, Ottersen G, Stenseth NC. Climate and the match or mismatch between predator requirements and resource availability. Clim Res. 2007;33:271–283.

[52] Doiron M, Gauthier G, Lévesque E. Trophic mismatch and its effects on the growth of young in an Arctic herbivore. Glob Chang Biol. 2015;21:4364–4376. doi: 10.1111/gcb.13057 26235037

[53] Ross MV, Alisauskas RT, Douglas DC, Kellett DK. Decadal declines in avian herbivore reproduction: density-dependent nutrition and phenological mismatch in the Arctic. Ecology. 2017;98:1869–1883. doi: 10.1002/ecy.1856 28403519

[54] Schwartz MD, Ahas R, Aasa A. Onset of spring starting earlier across the Northern Hemisphere Glob Chang Biol. 2006;12:343–351.

[55] Sparks TH, Aasa A, Huber K, Wadsworth R. Changes and patterns in biologically relevant temperatures in Europe 1941–2000. Clim Res. 2009;39:191–207.

[56] Monahan WB, Rosemartin A, Gerst KL, Fisichelli NA, Ault T, Schwartz MD, et al. Climate change is advancing spring onset across the U.S national park system. Ecosphere. 2016;7(10):e01465.

[57] Harrington R, Woiwod I, Sparks T. Climate change and trophic interactions. Trends Ecol Evol. 1999;14:146–150. doi: 10.1016/s0169-5347(99)01604-3 10322520

[58] Ge Q, Wang H, Rutishauser T, Dai J. Phenological response to climate change in China: a meta‐analysis. Glob Chang Biol. 2015;21:265–274. doi: 10.1111/gcb.12648 24895088

[59] Cohen JM, Lajeunesse MJ, Rohr JR. A global synthesis of animal phenological responses to climate change. Nat Clim Chang. 2018;8:224–228.

[60] Si Y, Xin Q, Prins HH, de Boer WF, Gong P. Improving the quantification of waterfowl migration with remote sensing and bird tracking. Sci Bull. 2015;60:1984–1993. doi: 10.1007/s11434-015-0930-9

[61] Schummer ML, Kaminski RM, Raedeke AH, Graber DA. Weather-related indices of autumn and winter dabbling duck abundance in middle North America. J Wildl Manage. 2010;74:94–101.

[62] Van Den Elsen LM. Weather and photoperiod indices of autumn and winter dabbling duck abundance in the Mississippi and Atlantic Flyways of North America. M.Sc. Thesis, London (ONT): University of Western Ontario; 2016.

[63] Xu F, Si Y. The frost wave hypothesis: how the environment drives autumn departure of migratory waterfowl. Ecol Indic. 2019;101:1018–1025. doi: 10.1016/j.ecolind.2019.02.024

[64] Smith TJ 3rd, Hayden BP. Snow Goose migration phenology is related to extratropical storm climate. Int J Biometeorol. 1984;28:225–233.

[65] Liechti F. Birds: blowin’ by the wind? J Ornithol. 2006;147:202–211.

[66] O’Neal BJ, Stafford JD, Larkin RP, Michel ES. The effect of weather on the decision to migrate from stopover sites by autumn-migrating ducks. Mov Ecol. 2018;6(23):1–9. doi: 10.1186/s40462-018-0141-5 30505448 PMC6257954

[67] Madsen J. Experimental refuges for migratory waterfowl in Danish wetlands. II. Tests of hunting disturbance effects. J Appl Ecol. 1998;35:398–417.

[68] Väänänen VM. Hunting disturbance and the timing of autumn migration in Anas species Wildlife Biol. 2001;7:3–9.

[69] Eichhorn G, Drent RH, Stahl J, Leito A, Alerstam T. Skipping the Baltic: the emergence of a dichotomy of alternative spring migration strategies in Russian Barnacle Geese. J Anim Ecol. 2009;78:63–72. doi: 10.1111/j.1365-2656.2008.01485.x 19120596

[70] Stirnemann RL, O’Halloran J, Ridgway M, Donnelly A. Temperature-related increases in grass growth and greater competition for food drive earlier migrational departure of wintering Whooper Swans. Ibis. 2012;154:542–553.

[71] Jonker RM, Eichhorn G, Langevelde FV, Bauer S. Predation danger can explain changes in timing of migration: the case of the Barnacle Goose. PLoS ONE. 2010;5(6):e11369. doi: 10.1371/journal.pone.0011369 20614027 PMC2894857

[72] Davis JB, Guillemain M, Kaminski RM, Arzel C, Eadie JM, Rees EC. Habitat and resource use by waterfowl in the Northern Hemisphere in autumn and winter. Wildfowl Special Issue. 2014;4:17–69.

[73] Møller AP, Fiedler W. Long-term time series of ornithological data. In: Møller AP, Fiedler W, Berthold P, editors. Effect of climate change on birds. London: Oxford University Press; 2010. pp. 33–38.

