# Supplementary material for: Changes in waterfowl migration phenologies in central North America: Implications for future waterfowl conservation
Source: PLoS One. 2022 May 18;17(5):e0266785. doi: 10.1371/journal.pone.0266785 (PMC9116660; doi:10.1371/journal.pone.0266785)
Supplement: S1 Table — (DOCX) [file pone.0266785.s001.docx]

**S1** **Table. Phenological changes in spring abundance peak across years for waterfowl at National Wildlife Refuges in the mid to lower portion of the Central Flyway (Nebraska, Kansas, Oklahoma, New Mexico, Texas), 1955–2008.** Results are from logistic regressions of spring peak month as a function of year for each possible species-refuge combination. We defined spring peak month as the month with the highest monthly count average within the January–March period. RL denotes the number of response levels for the dependent variable (i.e., peak month). Binomial data sets (i.e., when all peaks fell within 2 different months; RL = 2) were analyzed using logistic regression with penalized maximum likelihood estimation and trinomial data sets (i.e., when all 3 months were represented; RL = 3) by ordinal logistic regression with a proportional odds version of the cumulative logit model. An RL of 1 indicates identical values of peak month across all years. All probabilities modeled were cumulated over higher values of peak month; thus, positive slopes indicate delays in peak month over time and negative slopes advancements.

| Species and refuge | RL | *n* | β | 95% CI | χ^2^ | df | *P* |
| --- | --- | --- | --- | --- | --- | --- | --- |
| Snow Goose (*Anser caerulescens*) |  |  |  |  |  |  |  |
| Bitter Lake | 3 | 7 | 0.746 | 0.092–2.428 | 5.46 | 1 | 0.019 |
| Ross's Goose (*Anser rossii*) |  |  |  |  |  |  |  |
| Bitter Lake | 2 | 6 | 0.649 | -0.053–5.732 | 3.04 | 1 | 0.081 |
| Light geese (*Anser caerulescens*/*rossii*) |  |  |  |  |  |  |  |
| Anahuac^a^ | 2 | 19 | 0.099 | -0.054–0.280 | 1.57 | 1 | 0.21 |
| Aransas^b^ | 2 | 18 | -0.131 | -0.323–0.020 | 2.86 | 1 | 0.091 |
| Attwater Prairie Chicken | 3 | 11 | -0.121 | -0.372–0.084 | 1.31 | 1 | 0.25 |
| Big Boggy | 2 | 13 | 0.015 | -0.182–0.216 | 0.02 | 1 | 0.88 |
| Bosque del Apache | 2 | 19 | 0.046 | -0.044–0.201 | 0.88 | 1 | 0.35 |
| Brazoria | 2 | 15 | -0.002 | -0.227–0.206 | <0.001 | 1 | 0.98 |
| Flint Hills | 2 | 7 | -1.069 | -5.902–-0.071 | 4.65 | 1 | 0.031 |
| Laguna Atascosa | 2 | 12 | -0.166 | -0.667–0.131 | 1.12 | 1 | 0.29 |
| McFaddin^c^ | 2 | 19 | -0.104 | -0.286–0.044 | 1.85 | 1 | 0.17 |
| North Platte^d^ | 2 | 18 | 0.047 | -0.099–0.196 | 0.44 | 1 | 0.51 |
| Quivira | 3 | 29 | -0.104 | -0.184–-0.045 | 13.95 | 1 | <0.001 |
| Salt Plains | 3 | 35 | -0.109 | -0.172–-0.060 | 24.12 | 1 | <0.001 |
| San Bernard | 2 | 16 | -0.080 | -0.263–0.077 | 0.98 | 1 | 0.32 |
| Tishomingo | 1 | 6 | ^e^ | ^e^ | ^e^ | ^e^ | ^e^ |
| Washita | 2 | 14 | -0.115 | -0.395–0.044 | 1.87 | 1 | 0.17 |
| Greater White-fronted Goose (*Anser albifrons*) |  |  |  |  |  |  |  |
| Anahuac^a^ | 3 | 19 | -0.068 | -0.226–0.075 | 0.86 | 1 | 0.35 |
| Aransas^b^ | 2 | 14 | -0.028 | -0.230–0.160 | 0.09 | 1 | 0.77 |
| Flint Hills | 3 | 7 | -0.034 | -0.745–0.685 | 0.010 | 1 | 0.92 |
| Kirwin | 3 | 36 | -0.004 | -0.065–0.056 | 0.02 | 1 | 0.89 |
| McFaddin^c^ | 3 | 19 | 0.065 | -0.072–0.212 | 0.87 | 1 | 0.35 |
| Quivira | 3 | 30 | -0.050 | -0.100–-0.006 | 4.95 | 1 | 0.026 |
| Salt Plains | 3 | 35 | -0.049 | -0.089–-0.013 | 7.38 | 1 | 0.007 |
| Tishomingo | 3 | 6 | 0.334 | -0.599–1.481 | 0.49 | 1 | 0.48 |
| Washita | 2 | 14 | 0.013 | -0.109–0.138 | 0.04 | 1 | 0.83 |
| Canada geese (*Branta hutchinsii*/*canadensis*) |  |  |  |  |  |  |  |
| Anahuac^a^ | 3 | 16 | -0.186 | -0.435–0.019 | 3.13 | 1 | 0.077 |
| Aransas^b^ | 2 | 16 | 0.018 | -0.151–0.189 | 0.05 | 1 | 0.83 |
| Bitter Lake | 2 | 7 | 0.329 | -0.217–5.333 | 1.11 | 1 | 0.29 |
| Bosque del Apache | 3 | 18 | -0.022 | -0.120–0.075 | 0.23 | 1 | 0.63 |
| Crescent Lake | 2 | 10 | 0.176 | -0.341–1.135 | 0.41 | 1 | 0.52 |
| Flint Hills | 3 | 33 | 0.014 | -0.051–0.081 | 0.18 | 1 | 0.67 |
| Kirwin | 3 | 40 | -0.008 | -0.065–0.049 | 0.07 | 1 | 0.79 |
| North Platte^d^ | 3 | 23 | 0.032 | -0.071–0.139 | 0.37 | 1 | 0.54 |
| Quivira | 3 | 29 | -0.053 | -0.108–-0.005 | 4.82 | 1 | 0.028 |
| Salt Plains | 3 | 35 | -0.021 | -0.063–0.019 | 1.03 | 1 | 0.31 |
| San Bernard | 2 | 9 | 0.016 | -0.331–0.370 | 0.008 | 1 | 0.93 |
| Tishomingo | 3 | 6 | 0.043 | -1.055–1.143 | 0.007 | 1 | 0.93 |
| Washita | 1 | 14 | ^e^ | ^e^ | ^e^ | ^e^ | ^e^ |
| Blue-winged Teal (*Spatula discors*) |  |  |  |  |  |  |  |
| Anahuac^a^ | 3 | 19 | -0.189 | -0.841–0.097 | 1.57 | 1 | 0.21 |
| Aransas^b^ | 3 | 18 | -0.025 | -0.233–0.178 | 0.07 | 1 | 0.80 |
| Attwater Prairie Chicken | 3 | 13 | -0.068 | -0.247–0.094 | 0.67 | 1 | 0.41 |
| Big Boggy | 2 | 16 | -0.182 | -0.774–0.103 | 1.49 | 1 | 0.22 |
| Brazoria | 2 | 16 | -0.275 | -4.064–0.056 | 2.51 | 1 | 0.11 |
| Flint Hills | 1 | 7 | ^e^ | ^e^ | ^e^ | ^e^ | ^e^ |
| Laguna Atascosa | 2 | 16 | 0.065 | -0.101–0.268 | 0.57 | 1 | 0.45 |
| Matagorda Island | 2 | 18 | -0.015 | -0.306–0.279 | 0.01 | 1 | 0.91 |
| McFaddin^c^ | 2 | 19 | -0.093 | -0.462–0.180 | 0.47 | 1 | 0.49 |
| Quivira | 2 | 24 | -0.026 | -0.307–0.069 | 0.25 | 1 | 0.62 |
| Salt Plains | 2 | 34 | 0.043 | -0.042–0.240 | 0.96 | 1 | 0.33 |
| San Bernard | 1 | 16 | ^e^ | ^e^ | ^e^ | ^e^ | ^e^ |
| Texas Point | 2 | 22 | -0.112 | -0.608–0.146 | 0.69 | 1 | 0.41 |
| Cinnamon Teal (*Spatula cyanoptera*) |  |  |  |  |  |  |  |
| Bosque del Apache | 2 | 19 | 0.084 | -0.009–0.359 | 3.29 | 1 | 0.070 |
| Northern Shoveler (*Spatula clypeata*) |  |  |  |  |  |  |  |
| Anahuac^a^ | 3 | 19 | -0.185 | -0.371–-0.028 | 5.43 | 1 | 0.020 |
| Aransas^b^ | 3 | 18 | -0.200 | -0.417–-0.035 | 5.83 | 1 | 0.016 |
| Attwater Prairie Chicken | 3 | 13 | -0.145 | -0.357–0.029 | 2.62 | 1 | 0.11 |
| Big Boggy | 3 | 16 | 0.032 | -0.128–0.205 | 0.16 | 1 | 0.69 |
| Bitter Lake | 2 | 7 | 0.272 | -0.202–1.124 | 1.21 | 1 | 0.27 |
| Bosque del Apache | 3 | 19 | 0.205 | 0.068–0.439 | 9.72 | 1 | 0.002 |
| Brazoria | 3 | 16 | -0.230 | -0.482–-0.035 | 5.46 | 1 | 0.019 |
| Crescent Lake | 1 | 9 | ^e^ | ^e^ | ^e^ | ^e^ | ^e^ |
| Flint Hills | 1 | 7 | ^e^ | ^e^ | ^e^ | ^e^ | ^e^ |
| Kirwin | 1 | 24 | ^e^ | ^e^ | ^e^ | ^e^ | ^e^ |
| Laguna Atascosa | 3 | 16 | -0.160 | -0.345–0.000 | 3.83 | 1 | 0.050 |
| Matagorda Island | 3 | 18 | -0.308 | -0.576–-0.116 | 11.58 | 1 | <0.001 |
| McFaddin^c^ | 3 | 19 | -0.043 | -0.206–0.114 | 0.29 | 1 | 0.59 |
| Quivira | 2 | 27 | -0.033 | -0.313–0.063 | 0.39 | 1 | 0.53 |
| Salt Plains | 3 | 35 | 0.003 | -0.087–0.087 | 0.007 | 1 | 0.93 |
| San Bernard | 3 | 16 | -0.292 | -0.565–-0.090 | 8.69 | 1 | 0.003 |
| Texas Point | 3 | 22 | -0.015 | -0.197–0.161 | 0.03 | 1 | 0.86 |
| Gadwall (*Mareca strepera*) |  |  |  |  |  |  |  |
| Anahuac^a^ | 3 | 19 | -0.137 | -0.316–0.015 | 3.12 | 1 | 0.077 |
| Aransas^b^ | 3 | 18 | -0.119 | -0.331–0.045 | 1.95 | 1 | 0.16 |
| Attwater Prairie Chicken | 3 | 13 | -0.186 | -0.420–-0.001 | 3.88 | 1 | 0.049 |
| Big Boggy | 3 | 16 | 0.099 | -0.054–0.272 | 1.58 | 1 | 0.21 |
| Bosque del Apache | 3 | 19 | 0.207 | 0.062–0.553 | 9.39 | 1 | 0.002 |
| Brazoria | 3 | 16 | -0.031 | -0.217–0.143 | 0.12 | 1 | 0.73 |
| Crescent Lake | 1 | 9 | ^e^ | ^e^ | ^e^ | ^e^ | ^e^ |
| Flint Hills | 2 | 7 | 0.000 | -0.906–0.906 | <0.001 | 1 | 1.00 |
| Laguna Atascosa | 3 | 16 | -0.066 | -0.231–0.080 | 0.78 | 1 | 0.38 |
| Matagorda Island | 3 | 18 | -0.073 | -0.234–0.074 | 0.93 | 1 | 0.33 |
| McFaddin^c^ | 3 | 19 | -0.054 | -0.207–0.090 | 0.54 | 1 | 0.46 |
| Salt Plains | 3 | 35 | -0.020 | -0.065–0.021 | 0.89 | 1 | 0.35 |
| San Bernard | 3 | 16 | -0.185 | -0.403–-0.008 | 4.23 | 1 | 0.040 |
| Texas Point | 3 | 22 | 0.133 | 0.002–0.283 | 3.94 | 1 | 0.047 |
| Tishomingo | 1 | 5 | ^e^ | ^e^ | ^e^ | ^e^ | ^e^ |
| American Wigeon (*Mareca americana*) |  |  |  |  |  |  |  |
| Anahuac^a^ | 3 | 19 | -0.077 | -0.235–0.068 | 1.07 | 1 | 0.30 |
| Aransas^b^ | 3 | 18 | -0.013 | -0.157–0.128 | 0.04 | 1 | 0.85 |
| Attwater Prairie Chicken | 3 | 10 | -0.030 | -0.241–0.166 | 0.09 | 1 | 0.76 |
| Big Boggy | 3 | 16 | 0.125 | -0.039–0.309 | 2.23 | 1 | 0.14 |
| Brazoria | 3 | 16 | 0.052 | -0.128–0.241 | 0.32 | 1 | 0.57 |
| Crescent Lake | 1 | 9 | ^e^ | ^e^ | ^e^ | ^e^ | ^e^ |
| Kirwin | 2 | 24 | 0.215 | 0.017–0.618 | 4.72 | 1 | 0.030 |
| Laguna Atascosa | 3 | 16 | -0.031 | -0.183–0.114 | 0.17 | 1 | 0.68 |
| Matagorda Island | 3 | 18 | -0.030 | -0.201–0.127 | 0.14 | 1 | 0.71 |
| McFaddin^c^ | 3 | 19 | 0.037 | -0.111–0.193 | 0.24 | 1 | 0.62 |
| North Platte^d^ | 2 | 22 | -0.064 | -0.441–0.169 | 0.28 | 1 | 0.60 |
| Quivira | 3 | 24 | 0.012 | -0.058–0.081 | 0.12 | 1 | 0.73 |
| Salt Plains | 3 | 35 | 0.006 | -0.034–0.047 | 0.10 | 1 | 0.75 |
| San Bernard | 3 | 16 | 0.079 | -0.076–0.248 | 1.00 | 1 | 0.32 |
| Washita | 3 | 14 | 0.028 | -0.119–0.178 | 0.15 | 1 | 0.69 |
| Mallard (*Anas platyrhynchos*) |  |  |  |  |  |  |  |
| Anahuac^a^ | 2 | 19 | 0.092 | -0.060–0.270 | 1.38 | 1 | 0.24 |
| Bitter Lake | 1 | 7 | ^e^ | ^e^ | ^e^ | ^e^ | ^e^ |
| Bosque del Apache | 2 | 19 | 0.008 | -0.087–0.169 | 0.02 | 1 | 0.88 |
| Crescent Lake | 1 | 9 | ^e^ | ^e^ | ^e^ | ^e^ | ^e^ |
| Flint Hills | 3 | 35 | -0.005 | -0.067–0.056 | 0.03 | 1 | 0.87 |
| Kirwin | 3 | 24 | 0.051 | -0.058–0.171 | 0.84 | 1 | 0.36 |
| McFaddin^c^ | 2 | 19 | -0.247 | -0.736–0.004 | 3.70 | 1 | 0.054 |
| North Platte^d^ | 3 | 23 | 0.006 | -0.104–0.118 | 0.01 | 1 | 0.91 |
| Quivira | 3 | 27 | -0.019 | -0.065–0.026 | 0.69 | 1 | 0.40 |
| Salt Plains | 3 | 35 | 0.006 | -0.035–0.051 | 0.09 | 1 | 0.77 |
| Tishomingo | 2 | 5 | 0.901 | -0.441–6.492 | 1.52 | 1 | 0.22 |
| Washita | 2 | 14 | -0.095 | -0.415–0.078 | 1.04 | 1 | 0.31 |
| Northern Pintail (*Anas acuta*) |  |  |  |  |  |  |  |
| Anahuac^a^ | 2 | 19 | 0.000 | -0.179–0.172 | <0.001 | 1 | 1.00 |
| Aransas^b^ | 2 | 18 | -0.153 | -0.961–0.132 | 0.96 | 1 | 0.33 |
| Attwater Prairie Chicken | 2 | 13 | -0.105 | -0.376–0.081 | 1.15 | 1 | 0.28 |
| Big Boggy | 2 | 16 | 0.020 | -0.162–0.202 | 0.05 | 1 | 0.82 |
| Bitter Lake | 1 | 7 | ^e^ | ^e^ | ^e^ | ^e^ | ^e^ |
| Bosque del Apache | 2 | 19 | -0.006 | -0.087–0.082 | 0.02 | 1 | 0.89 |
| Brazoria | 2 | 16 | 0.036 | -0.137–0.216 | 0.17 | 1 | 0.68 |
| Flint Hills | 1 | 7 | ^e^ | ^e^ | ^e^ | ^e^ | ^e^ |
| Kirwin | 2 | 24 | 0.049 | -0.059–0.166 | 0.78 | 1 | 0.38 |
| Laguna Atascosa | 2 | 16 | -0.149 | -0.567–0.078 | 1.50 | 1 | 0.22 |
| Matagorda Island | 2 | 18 | -0.097 | -0.320–0.075 | 1.17 | 1 | 0.28 |
| McFaddin^c^ | 2 | 18 | 0.029 | -0.140–0.200 | 0.12 | 1 | 0.73 |
| North Platte^d^ | 2 | 22 | -0.060 | -0.438–0.166 | 0.25 | 1 | 0.62 |
| Quivira | 2 | 27 | -0.066 | -0.132–-0.016 | 6.92 | 1 | 0.009 |
| Salt Plains | 3 | 35 | -0.028 | -0.067–0.009 | 2.23 | 1 | 0.14 |
| San Bernard | 2 | 16 | 0.020 | -0.143–0.185 | 0.06 | 1 | 0.81 |
| Tishomingo | 3 | 5 | 0.752 | -0.525–2.617 | 1.30 | 1 | 0.25 |
| Washita | 3 | 14 | 0.001 | -0.129–0.130 | <0.001 | 1 | 0.99 |
| Green-winged Teal (*Anas crecca*) |  |  |  |  |  |  |  |
| Anahuac^a^ | 2 | 19 | 0.079 | -0.072–0.252 | 1.04 | 1 | 0.31 |
| Aransas^b^ | 3 | 18 | -0.156 | -0.350–0.001 | 3.81 | 1 | 0.051 |
| Attwater Prairie Chicken | 3 | 12 | -0.174 | -0.498–0.053 | 2.15 | 1 | 0.14 |
| Big Boggy | 3 | 16 | -0.113 | -0.328–0.063 | 1.54 | 1 | 0.21 |
| Bitter Lake | 3 | 6 | 0.101 | -0.428–0.708 | 0.15 | 1 | 0.70 |
| Bosque del Apache | 3 | 19 | -0.013 | -0.136–0.078 | 0.07 | 1 | 0.79 |
| Brazoria | 3 | 16 | -0.074 | -0.296–0.117 | 0.56 | 1 | 0.46 |
| Crescent Lake | 1 | 9 | ^e^ | ^e^ | ^e^ | ^e^ | ^e^ |
| Flint Hills | 3 | 7 | -0.469 | -1.765–0.423 | 1.02 | 1 | 0.31 |
| Kirwin | 2 | 22 | -0.012 | -0.251–0.216 | 0.01 | 1 | 0.90 |
| Laguna Atascosa | 3 | 16 | 0.007 | -0.167–0.180 | 0.006 | 1 | 0.94 |
| Matagorda Island | 3 | 18 | -0.087 | -0.252–0.060 | 1.32 | 1 | 0.25 |
| McFaddin^c^ | 3 | 19 | -0.052 | -0.200–0.090 | 0.52 | 1 | 0.47 |
| Quivira | 3 | 26 | -0.022 | -0.108–0.044 | 0.40 | 1 | 0.53 |
| Salt Plains | 3 | 35 | -0.011 | -0.056–0.030 | 0.27 | 1 | 0.61 |
| San Bernard | 3 | 16 | -0.192 | -0.418–-0.016 | 4.63 | 1 | 0.031 |
| Texas Point | 3 | 22 | -0.226 | -0.448–-0.062 | 7.91 | 1 | 0.005 |
| Tishomingo | 3 | 5 | 0.274 | -0.883–1.672 | 0.22 | 1 | 0.64 |
| Washita | 3 | 12 | -0.101 | -0.294–0.061 | 1.46 | 1 | 0.23 |
| Canvasback (*Aythya valisineria*) |  |  |  |  |  |  |  |
| Aransas^b^ | 2 | 17 | -0.054 | -0.224–0.099 | 0.48 | 1 | 0.49 |
| Brazoria | 2 | 14 | 0.024 | -0.156–0.213 | 0.07 | 1 | 0.79 |
| Crescent Lake | 1 | 9 | ^e^ | ^e^ | ^e^ | ^e^ | ^e^ |
| Laguna Atascosa | 2 | 14 | -0.239 | -0.918–0.052 | 2.34 | 1 | 0.13 |
| McFaddin^c^ | 2 | 16 | -0.089 | -0.333–0.096 | 0.83 | 1 | 0.36 |
| Redhead (*Aythya americana*) |  |  |  |  |  |  |  |
| Aransas^b^ | 3 | 18 | -0.080 | -0.249–0.068 | 1.11 | 1 | 0.29 |
| Brazoria | 3 | 16 | -0.151 | -0.377–0.032 | 2.58 | 1 | 0.11 |
| Crescent Lake | 1 | 9 | ^e^ | ^e^ | ^e^ | ^e^ | ^e^ |
| Kirwin | 2 | 24 | 0.009 | -0.118–0.139 | 0.02 | 1 | 0.88 |
| Laguna Atascosa | 2 | 16 | -0.045 | -0.207–0.102 | 0.37 | 1 | 0.55 |
| Matagorda Island | 2 | 18 | -0.032 | -0.194–0.118 | 0.18 | 1 | 0.68 |
| North Platte^d^ | 1 | 22 | ^e^ | ^e^ | ^e^ | ^e^ | ^e^ |
| Quivira | 2 | 27 | -0.070 | -0.272–0.014 | 2.45 | 1 | 0.12 |
| Salt Plains | 3 | 34 | -0.064 | -0.124–-0.018 | 7.98 | 1 | 0.005 |
| Washita | 3 | 13 | -0.047 | -0.185–0.079 | 0.54 | 1 | 0.46 |
| Ring-necked Duck (*Aythya collaris*) |  |  |  |  |  |  |  |
| Flint Hills | 2 | 7 | 0.163 | -0.669–1.253 | 0.16 | 1 | 0.69 |
| Scaup (*Aythya marila*/*affinis*) |  |  |  |  |  |  |  |
| Anahuac^a^ | 3 | 19 | 0.088 | -0.057–0.249 | 1.40 | 1 | 0.24 |
| Aransas^b^ | 3 | 18 | 0.012 | -0.125–0.148 | 0.03 | 1 | 0.86 |
| Big Boggy | 3 | 15 | -0.268 | -0.614–-0.040 | 5.60 | 1 | 0.018 |
| Crescent Lake | 1 | 9 | ^e^ | ^e^ | ^e^ | ^e^ | ^e^ |
| Kirwin | 2 | 20 | 0.015 | -0.165–0.207 | 0.03 | 1 | 0.87 |
| Laguna Atascosa | 3 | 16 | 0.064 | -0.076–0.215 | 0.80 | 1 | 0.37 |
| Matagorda Island | 3 | 18 | -0.109 | -0.271–0.036 | 2.14 | 1 | 0.14 |
| McFaddin^c^ | 2 | 18 | -0.011 | -0.166–0.140 | 0.02 | 1 | 0.88 |
| Quivira | 2 | 27 | -0.033 | -0.313–0.063 | 0.39 | 1 | 0.53 |
| Salt Plains | 3 | 35 | -0.067 | -0.128–-0.021 | 8.92 | 1 | 0.003 |
| Texas Point | 3 | 22 | 0.025 | -0.106–0.158 | 0.15 | 1 | 0.70 |
| Bufflehead (*Bucephala albeola*) |  |  |  |  |  |  |  |
| Quivira | 3 | 26 | -0.216 | -0.812–-0.002 | 3.96 | 1 | 0.047 |
| Goldeneyes (*Bucephala clangula*/*islandica*) |  |  |  |  |  |  |  |
| Kirwin | 3 | 24 | 0.065 | -0.049–0.188 | 1.22 | 1 | 0.27 |
| Common Merganser (*Mergus merganser*) |  |  |  |  |  |  |  |
| Flint Hills | 2 | 7 | -0.215 | -1.008–0.433 | 0.42 | 1 | 0.52 |
| Kirwin | 3 | 24 | 0.086 | -0.014–0.197 | 2.82 | 1 | 0.093 |
| North Platte^d^ | 3 | 22 | 0.006 | -0.164–0.169 | 0.005 | 1 | 0.94 |
| Quivira | 3 | 26 | -0.021 | -0.070–0.025 | 0.82 | 1 | 0.36 |
| Salt Plains | 2 | 35 | 0.021 | -0.016–0.062 | 1.23 | 1 | 0.27 |
| Ruddy Duck (*Oxyura jamaicensis*) |  |  |  |  |  |  |  |
| Bitter Lake | 2 | 7 | -0.013 | -0.598–0.466 | 0.003 | 1 | 0.96 |
| Bosque del Apache | 3 | 19 | 0.098 | -0.018–0.235 | 2.79 | 1 | 0.095 |
| Quivira | 1 | 25 | ^e^ | ^e^ | ^e^ | ^e^ | ^e^ |
| Salt Plains | 3 | 34 | -0.029 | -0.088–0.019 | 1.34 | 1 | 0.25 |

^a^ Several new areas were added to the Anahuac surveys over time.

^b^ One new area was added to the Aransas surveys from October 1988 onward.

^c^ Several new areas were added to the McFaddin surveys over time.

^d^ One new area was added to the North Platte surveys from October 1991 onward.

^e^ No regression performed due to zero variation in the dependent variable (i.e., peak month).
