# Supplementary material for: Changes in waterfowl migration phenologies in central North America: Implications for future waterfowl conservation
Source: PLoS One. 2022 May 18;17(5):e0266785. doi: 10.1371/journal.pone.0266785 (PMC9116660; doi:10.1371/journal.pone.0266785)
Supplement: S4 Table — (DOCX) [file pone.0266785.s004.docx]

**S4 Table. Phenological changes in autumn abundance peak in relation to temperature for waterfowl at National Wildlife Refuges in the mid to lower portion of the Central Flyway (Nebraska, Kansas, Oklahoma, New Mexico, Texas), 1955–2008.** Results are from logistic regressions of autumn peak month as a function of average daily maximum temperature for October–December for each possible species-refuge combination. We defined autumn peak month as the month with the highest monthly count average within the October–December period. RL denotes the number of response levels for the dependent variable (i.e., peak month). Binomial data sets (i.e., when all peaks fell within 2 different months; RL = 2) were analyzed using logistic regression with penalized maximum likelihood estimation and trinomial data sets (i.e., when all 3 months were represented; RL = 3) by ordinal logistic regression with a proportional odds version of the cumulative logit model. An RL of 1 indicates identical values of peak month across all temperature averages. All probabilities modeled were cumulated over higher values of peak month; thus, positive slopes indicate delays in peak month with increasing temperature and negative slopes advancements.

| Species and refuge | RL | *n* | β | 95% CI | χ^2^ | df | *P* |
| --- | --- | --- | --- | --- | --- | --- | --- |
| Snow Goose (*Anser caerulescens*) |  |  |  |  |  |  |  |
| Bitter Lake | 3 | 9 | 1.764 | 0.259–7.055 | 6.30 | 1 | 0.012 |
| Ross's Goose (*Anser rossii*) |  |  |  |  |  |  |  |
| Bitter Lake | 3 | 8 | -0.339 | -1.544–0.556 | 0.53 | 1 | 0.47 |
| Light geese (*Anser caerulescens*/*rossii*) |  |  |  |  |  |  |  |
| Anahuac^a^ | 2 | 21 | -0.246 | -1.224–0.683 | 0.27 | 1 | 0.60 |
| Aransas^b^ | 2 | 20 | 0.419 | -0.37–1.303 | 1.07 | 1 | 0.30 |
| Big Boggy | 2 | 17 | -0.757 | -2.044–0.19 | 2.38 | 1 | 0.12 |
| Bosque del Apache | 2 | 19 | 0.503 | -0.171–1.279 | 2.11 | 1 | 0.15 |
| Brazoria | 2 | 20 | 0.079 | -0.84–0.972 | 0.03 | 1 | 0.86 |
| Flint Hills | 2 | 6 | 0.744 | -0.487–4.161 | 1.29 | 1 | 0.26 |
| Laguna Atascosa | 2 | 20 | 1.837 | 0.515–3.977 | 8.95 | 1 | 0.003 |
| McFaddin^c^ | 2 | 21 | -0.158 | -1.323–0.892 | 0.09 | 1 | 0.77 |
| Quivira | 3 | 28 | -0.213 | -0.715–0.251 | 0.80 | 1 | 0.37 |
| Salt Plains | 3 | 33 | -0.554 | -1.17–-0.028 | 4.29 | 1 | 0.038 |
| San Bernard | 3 | 21 | 0.513 | -0.326–1.44 | 1.43 | 1 | 0.23 |
| Tishomingo | 2 | 6 | 0.302 | -0.424–1.595 | 0.64 | 1 | 0.42 |
| Washita | 2 | 18 | 0.292 | -0.549–1.261 | 0.47 | 1 | 0.49 |
| Greater White-fronted Goose (*Anser albifrons*) |  |  |  |  |  |  |  |
| Anahuac^a^ | 3 | 21 | -0.025 | -0.936–0.883 | 0.00 | 1 | 0.96 |
| Aransas^b^ | 3 | 19 | 0.224 | -0.538–1.02 | 0.33 | 1 | 0.56 |
| Brazoria | 3 | 9 | -0.225 | -2.045–1.492 | 0.07 | 1 | 0.79 |
| Flint Hills | 2 | 6 | 0.076 | -1.631–1.618 | 0.01 | 1 | 0.91 |
| Kirwin | 3 | 45 | 0.068 | -0.282–0.42 | 0.15 | 1 | 0.70 |
| Laguna Atascosa | 3 | 18 | 0.271 | -0.613–1.219 | 0.36 | 1 | 0.55 |
| McFaddin^c^ | 2 | 18 | 1.407 | 0.207–3.071 | 5.47 | 1 | 0.019 |
| Quivira | 3 | 28 | 0.230 | -0.195–0.685 | 1.11 | 1 | 0.29 |
| Salt Plains | 3 | 33 | -0.446 | -0.984–0.039 | 3.23 | 1 | 0.072 |
| San Bernard | 3 | 16 | -0.329 | -1.311–0.586 | 0.50 | 1 | 0.48 |
| Tishomingo | 2 | 5 | 0.526 | -0.452–5.638 | 0.76 | 1 | 0.38 |
| Washita | 2 | 18 | -0.373 | -1.188–0.283 | 1.21 | 1 | 0.27 |
| Canada geese (*Branta hutchinsii*/*canadensis*) |  |  |  |  |  |  |  |
| Aransas^b^ | 2 | 19 | -0.065 | -0.914–0.745 | 0.03 | 1 | 0.87 |
| Bitter Lake | 1 | 9 | ^e^ | ^e^ | ^e^ | ^e^ | ^e^ |
| Bosque del Apache | 2 | 19 | -0.003 | -0.794–0.804 | 0.00 | 1 | 0.99 |
| Brazoria | 3 | 8 | 0.111 | -1.427–1.661 | 0.02 | 1 | 0.88 |
| Crescent Lake | 3 | 9 | 1.577 | 0.315–4.221 | 7.09 | 1 | 0.008 |
| Flint Hills | 2 | 34 | -0.121 | -0.588–0.31 | 0.30 | 1 | 0.58 |
| Kirwin | 3 | 47 | 0.574 | 0.145–1.093 | 7.07 | 1 | 0.008 |
| North Platte^d^ | 2 | 25 | 0.202 | -0.249–0.723 | 0.76 | 1 | 0.38 |
| Quivira | 3 | 28 | 0.300 | -0.295–0.990 | 0.94 | 1 | 0.33 |
| Salt Plains | 2 | 33 | -0.071 | -0.589–0.419 | 0.08 | 1 | 0.78 |
| Tishomingo | 1 | 6 | ^e^ | ^e^ | ^e^ | ^e^ | ^e^ |
| Washita | 2 | 18 | 0.131 | -0.639–0.949 | 0.12 | 1 | 0.73 |
| Wood Duck (*Aix sponsa*) |  |  |  |  |  |  |  |
| Deep Fork | 2 | 6 | -0.280 | -1.869–1.163 | 0.15 | 1 | 0.70 |
| Blue-winged Teal (*Spatula discors*) |  |  |  |  |  |  |  |
| Anahuac^a^ | 3 | 22 | -0.087 | -1.048–0.894 | 0.03 | 1 | 0.86 |
| Aransas^b^ | 3 | 20 | 1.071 | -0.079–2.824 | 3.27 | 1 | 0.071 |
| Attwater Prairie Chicken |  | 17 | -0.462 | -1.355–0.314 | 1.34 | 1 | 0.25 |
| Big Boggy | 3 | 18 | 0.159 | -0.776–1.204 | 0.11 | 1 | 0.74 |
| Brazoria | 2 | 20 | 1.242 | -0.5–6.451 | 1.55 | 1 | 0.21 |
| Flint Hills | 1 | 6 | ^e^ | ^e^ | ^e^ | ^e^ | ^e^ |
| Laguna Atascosa | 2 | 20 | 0.230 | -1.049–2.113 | 0.11 | 1 | 0.74 |
| Matagorda Island | 3 | 21 | 0.926 | -0.404–2.887 | 1.74 | 1 | 0.19 |
| McFaddin^c^ | 3 | 21 | 0.448 | -0.577–1.608 | 0.72 | 1 | 0.40 |
| Quivira | 2 | 20 | -0.184 | -0.963–0.501 | 0.27 | 1 | 0.60 |
| Salt Plains | 2 | 24 | 0.176 | -0.937–1.462 | 0.09 | 1 | 0.77 |
| San Bernard | 2 | 21 | -0.361 | -1.432–0.663 | 0.51 | 1 | 0.47 |
| Texas Point | 3 | 16 | 0.174 | -0.949–1.38 | 0.09 | 1 | 0.76 |
| Cinnamon Teal (*Spatula cyanoptera*) |  |  |  |  |  |  |  |
| Bosque del Apache | 2 | 17 | -0.493 | -4.271–0.795 | 0.48 | 1 | 0.49 |
| Northern Shoveler (*Spatula clypeata*) |  |  |  |  |  |  |  |
| Anahuac^a^ | 3 | 22 | -0.935 | -2.376–0.187 | 2.61 | 1 | 0.11 |
| Aransas^b^ | 3 | 20 | -0.147 | -1.01–0.694 | 0.12 | 1 | 0.73 |
| Attwater Prairie Chicken |  | 19 | -0.468 | -1.382–0.307 | 1.37 | 1 | 0.24 |
| Big Boggy | 3 | 19 | 0.099 | -0.796–0.986 | 0.05 | 1 | 0.82 |
| Bitter Lake | 3 | 9 | -0.650 | -2.64–0.311 | 1.58 | 1 | 0.21 |
| Bosque del Apache | 3 | 18 | -0.366 | -1.141–0.343 | 1.02 | 1 | 0.31 |
| Brazoria | 3 | 20 | 0.222 | -0.665–1.172 | 0.24 | 1 | 0.62 |
| Crescent Lake | 2 | 9 | 0.036 | -0.708–1.733 | 0.01 | 1 | 0.93 |
| Flint Hills | 2 | 6 | -0.496 | -2.422–0.675 | 0.66 | 1 | 0.42 |
| Laguna Atascosa | 3 | 20 | 0.514 | -0.325–1.429 | 1.43 | 1 | 0.23 |
| Matagorda Island | 3 | 21 | -0.824 | -1.818–0.03 | 3.57 | 1 | 0.059 |
| McFaddin^c^ | 3 | 21 | -0.117 | -1.197–0.921 | 0.05 | 1 | 0.82 |
| Quivira | 3 | 19 | -0.202 | -0.832–0.385 | 0.46 | 1 | 0.50 |
| Salt Plains | 3 | 24 | 0.048 | -0.68–0.761 | 0.02 | 1 | 0.89 |
| San Bernard | 3 | 21 | -0.362 | -1.263–0.447 | 0.76 | 1 | 0.38 |
| Texas Point | 3 | 20 | -0.124 | -1.121–0.873 | 0.06 | 1 | 0.80 |
| Tishomingo | 2 | 5 | -0.048 | -0.899–0.995 | 0.01 | 1 | 0.91 |
| Gadwall (*Mareca strepera*) |  |  |  |  |  |  |  |
| Anahuac^a^ | 3 | 22 | 0.157 | -0.838–1.165 | 0.10 | 1 | 0.75 |
| Aransas^b^ | 3 | 20 | -2.045 | -3.856–-0.8 | 12.73 | 1 | <0.001 |
| Attwater Prairie Chicken |  | 19 | 0.005 | -0.916–0.898 | 0.00 | 1 | 0.99 |
| Big Boggy | 3 | 19 | -0.308 | -1.348–0.625 | 0.41 | 1 | 0.52 |
| Bosque del Apache | 3 | 18 | 0.277 | -0.519–1.188 | 0.46 | 1 | 0.50 |
| Brazoria | 3 | 20 | -0.583 | -1.645–0.333 | 1.52 | 1 | 0.22 |
| Crescent Lake | 1 | 8 | ^e^ | ^e^ | ^e^ | ^e^ | ^e^ |
| Flint Hills | 3 | 6 | 0.953 | -0.662–2.9 | 1.39 | 1 | 0.24 |
| Kirwin | 3 | 27 | 0.049 | -0.324–0.445 | 0.06 | 1 | 0.80 |
| Laguna Atascosa | 3 | 20 | -0.056 | -1.105–0.892 | 0.01 | 1 | 0.91 |
| Matagorda Island | 3 | 21 | -0.004 | -0.9–0.871 | 0.00 | 1 | 0.99 |
| McFaddin^c^ | 2 | 21 | -0.251 | -1.307–0.724 | 0.25 | 1 | 0.61 |
| Quivira | 3 | 20 | 0.162 | -0.495–0.848 | 0.24 | 1 | 0.63 |
| Salt Plains | 3 | 23 | 0.375 | -0.354–1.203 | 1.00 | 1 | 0.32 |
| San Bernard | 3 | 21 | -0.358 | -1.294–0.469 | 0.71 | 1 | 0.40 |
| Texas Point | 3 | 21 | -1.701 | -3.539–-0.42 | 7.35 | 1 | 0.007 |
| Tishomingo | 2 | 6 | -0.045 | -0.829–0.813 | 0.02 | 1 | 0.90 |
| American Wigeon (*Mareca americana*) |  |  |  |  |  |  |  |
| Anahuac^a^ | 3 | 22 | -0.954 | -2.096–0.034 | 3.57 | 1 | 0.059 |
| Aransas^b^ | 3 | 20 | 0.049 | -0.75–0.859 | 0.01 | 1 | 0.90 |
| Attwater Prairie Chicken |  | 15 | -0.255 | -1.231–0.602 | 0.34 | 1 | 0.56 |
| Big Boggy | 3 | 18 | -0.252 | -1.226–0.69 | 0.28 | 1 | 0.59 |
| Bitter Lake | 2 | 9 | 0.346 | -0.383–1.195 | 0.86 | 1 | 0.35 |
| Bosque del Apache | 3 | 18 | -0.246 | -1–0.47 | 0.46 | 1 | 0.50 |
| Brazoria | 3 | 20 | -0.213 | -1.072–0.606 | 0.26 | 1 | 0.61 |
| Crescent Lake | 1 | 8 | ^e^ | ^e^ | ^e^ | ^e^ | ^e^ |
| Kirwin | 3 | 29 | 0.280 | -0.09–0.695 | 2.18 | 1 | 0.14 |
| Laguna Atascosa | 3 | 20 | -0.206 | -1.181–0.712 | 0.19 | 1 | 0.66 |
| Matagorda Island | 2 | 21 | 0.090 | -0.709–0.901 | 0.05 | 1 | 0.82 |
| McFaddin^c^ | 3 | 20 | -0.202 | -1.207–0.755 | 0.17 | 1 | 0.68 |
| North Platte^d^ | 2 | 24 | 0.231 | -0.758–1.258 | 0.17 | 1 | 0.68 |
| Quivira | 3 | 20 | 5.416 | 1.669–16.198 | 18.02 | 1 | <0.001 |
| Salt Plains | 3 | 24 | -0.172 | -0.837–0.472 | 0.28 | 1 | 0.59 |
| San Bernard | 3 | 21 | -1.029 | -2.033–-0.204 | 6.11 | 1 | 0.013 |
| Texas Point | 3 | 19 | -1.729 | -3.213–-0.543 | 8.65 | 1 | 0.003 |
| Tishomingo | 3 | 6 | 0.071 | -0.727–0.895 | 0.03 | 1 | 0.85 |
| Washita | 3 | 18 | -0.255 | -0.946–0.399 | 0.60 | 1 | 0.44 |
| Mallard (*Anas platyrhynchos*) |  |  |  |  |  |  |  |
| Anahuac^a^ | 2 | 22 | 2.012 | 0.592–4.476 | 8.84 | 1 | 0.003 |
| Bitter Lake | 2 | 9 | -0.156 | -2.012–0.803 | 0.10 | 1 | 0.75 |
| Bosque del Apache | 2 | 18 | -0.123 | -0.833–0.568 | 0.13 | 1 | 0.72 |
| Crescent Lake | 1 | 9 | ^e^ | ^e^ | ^e^ | ^e^ | ^e^ |
| Deep Fork | 2 | 6 | 0.278 | -1.243–1.865 | 0.14 | 1 | 0.71 |
| Flint Hills | 2 | 34 | 0.249 | -0.166–0.707 | 1.37 | 1 | 0.24 |
| Kirwin | 3 | 29 | 0.942 | 0.367–1.768 | 12.56 | 1 | <0.001 |
| McFaddin^c^ | 2 | 21 | 0.727 | -0.331–1.952 | 1.80 | 1 | 0.18 |
| North Platte^d^ | 2 | 25 | 0.250 | -0.179–0.788 | 1.26 | 1 | 0.26 |
| Quivira | 2 | 20 | -0.120 | -0.798–0.524 | 0.14 | 1 | 0.71 |
| Salt Plains | 3 | 24 | 0.634 | -0.093–1.479 | 2.89 | 1 | 0.089 |
| Tishomingo | 2 | 6 | 0.507 | -0.265–2.278 | 1.58 | 1 | 0.21 |
| Washita | 2 | 18 | 0.414 | -0.566–1.605 | 0.68 | 1 | 0.41 |
| Northern Pintail (*Anas acuta*) |  |  |  |  |  |  |  |
| Anahuac^a^ | 3 | 22 | 0.233 | -0.755–1.235 | 0.22 | 1 | 0.64 |
| Aransas^b^ | 2 | 20 | -0.159 | -1.039–0.656 | 0.15 | 1 | 0.70 |
| Attwater Prairie Chicken |  | 19 | 0.075 | -0.709–0.869 | 0.04 | 1 | 0.85 |
| Big Boggy | 3 | 18 | 0.889 | -0.008–1.956 | 3.77 | 1 | 0.052 |
| Bitter Lake | 2 | 9 | -0.510 | -2.068–0.292 | 1.46 | 1 | 0.23 |
| Bosque del Apache | 3 | 18 | 0.018 | -0.788–0.817 | 0.00 | 1 | 0.96 |
| Brazoria | 3 | 20 | -0.657 | -1.663–0.222 | 2.12 | 1 | 0.15 |
| Crescent Lake | 1 | 9 | ^e^ | ^e^ | ^e^ | ^e^ | ^e^ |
| Flint Hills | 2 | 6 | -1.837 | -5.912–0.431 | 2.05 | 1 | 0.15 |
| Kirwin | 3 | 28 | 0.221 | -0.162–0.644 | 1.27 | 1 | 0.26 |
| Laguna Atascosa | 3 | 20 | 0.338 | -0.767–1.432 | 0.40 | 1 | 0.53 |
| Matagorda Island | 3 | 21 | 0.246 | -0.729–1.243 | 0.26 | 1 | 0.61 |
| McFaddin^c^ | 3 | 21 | -0.001 | -1.134–1.08 | 0.00 | 1 | 1.00 |
| Quivira | 3 | 20 | 0.092 | -0.489–0.713 | 0.10 | 1 | 0.76 |
| Salt Plains | 3 | 24 | -0.125 | -0.88–0.593 | 0.12 | 1 | 0.73 |
| San Bernard | 3 | 20 | -0.027 | -0.764–0.726 | 0.01 | 1 | 0.94 |
| Tishomingo | 3 | 6 | -0.415 | -1.466–0.415 | 0.94 | 1 | 0.33 |
| Green-winged Teal (*Anas crecca*) |  |  |  |  |  |  |  |
| Anahuac^a^ | 2 | 22 | 0.208 | -0.818–1.243 | 0.17 | 1 | 0.68 |
| Aransas^b^ | 3 | 20 | 0.722 | -0.127–1.71 | 2.75 | 1 | 0.097 |
| Attwater Prairie Chicken |  | 18 | -0.119 | -0.935–0.653 | 0.09 | 1 | 0.76 |
| Big Boggy | 3 | 19 | 0.129 | -0.734–0.994 | 0.09 | 1 | 0.76 |
| Bitter Lake | 2 | 5 | 0.492 | -0.43–1.859 | 1.05 | 1 | 0.31 |
| Bosque del Apache | 3 | 18 | 0.229 | -0.487–0.991 | 0.39 | 1 | 0.53 |
| Brazoria | 3 | 20 | -0.428 | -1.504–0.498 | 0.79 | 1 | 0.37 |
| Crescent Lake | 1 | 9 | ^e^ | ^e^ | ^e^ | ^e^ | ^e^ |
| Flint Hills | 3 | 6 | -0.804 | -2.824–0.663 | 1.12 | 1 | 0.29 |
| Kirwin | 3 | 29 | 0.244 | -0.198–0.785 | 1.11 | 1 | 0.29 |
| Laguna Atascosa | 3 | 20 | -0.388 | -1.599–0.598 | 0.56 | 1 | 0.45 |
| Matagorda Island | 3 | 21 | 0.216 | -0.487–0.942 | 0.36 | 1 | 0.55 |
| McFaddin^c^ | 2 | 21 | 0.141 | -0.946–1.209 | 0.07 | 1 | 0.79 |
| Quivira | 3 | 20 | -0.031 | -0.597–0.522 | 0.01 | 1 | 0.91 |
| Salt Plains | 3 | 24 | 0.275 | -0.393–1.005 | 0.65 | 1 | 0.42 |
| San Bernard | 3 | 21 | 0.022 | -0.722–0.753 | 0.00 | 1 | 0.95 |
| Texas Point | 3 | 18 | -1.531 | -3.787–-0.036 | 4.06 | 1 | 0.044 |
| Tishomingo | 3 | 6 | 0.379 | -0.575–1.488 | 0.61 | 1 | 0.43 |
| Canvasback (*Aythya valisineria*) |  |  |  |  |  |  |  |
| Aransas^b^ | 3 | 20 | -0.763 | -1.854–0.121 | 2.82 | 1 | 0.093 |
| Crescent Lake | 1 | 9 | ^e^ | ^e^ | ^e^ | ^e^ | ^e^ |
| Laguna Atascosa | 3 | 18 | -0.348 | -1.405–0.619 | 0.50 | 1 | 0.48 |
| McFaddin^c^ | 3 | 19 | 0.292 | -0.778–1.412 | 0.29 | 1 | 0.59 |
| Quivira | 3 | 19 | -0.242 | -0.972–0.440 | 0.49 | 1 | 0.48 |
| Redhead (*Aythya americana*) |  |  |  |  |  |  |  |
| Aransas^b^ | 2 | 20 | 0.155 | -0.701–1.024 | 0.13 | 1 | 0.72 |
| Brazoria | 3 | 17 | -0.425 | -1.684–0.619 | 0.61 | 1 | 0.43 |
| Crescent Lake | 1 | 9 | ^e^ | ^e^ | ^e^ | ^e^ | ^e^ |
| Kirwin | 3 | 26 | 0.498 | 0.026–1.074 | 4.30 | 1 | 0.038 |
| Laguna Atascosa | 3 | 20 | -0.061 | -0.957–0.822 | 0.02 | 1 | 0.89 |
| Matagorda Island | 2 | 21 | 0.942 | -0.019–2.202 | 3.68 | 1 | 0.055 |
| North Platte^d^ | 2 | 24 | 0.297 | -0.904–1.288 | 0.30 | 1 | 0.58 |
| Quivira | 2 | 20 | 0.220 | -0.340–0.873 | 0.58 | 1 | 0.44 |
| Salt Plains | 3 | 24 | -0.172 | -0.915–0.51 | 0.24 | 1 | 0.62 |
| Tishomingo | 2 | 5 | 0.147 | -0.601–1.001 | 0.17 | 1 | 0.68 |
| Washita | 2 | 18 | -0.132 | -0.841–0.528 | 0.16 | 1 | 0.69 |
| Ring-necked Duck (*Aythya collaris*) |  |  |  |  |  |  |  |
| Flint Hills | 3 | 5 | -0.908 | -3.067–0.505 | 1.52 | 1 | 0.22 |
| North Platte^d^ | 2 | 25 | -0.292 | -0.858–0.168 | 1.51 | 1 | 0.22 |
| Tishomingo | 3 | 6 | 0.065 | -0.731–0.843 | 0.03 | 1 | 0.86 |
| Scaup (*Aythya marila*/*affinis*) |  |  |  |  |  |  |  |
| Anahuac^a^ | 2 | 21 | 1.013 | -0.02–2.273 | 3.68 | 1 | 0.055 |
| Aransas^b^ | 2 | 20 | 0.943 | 0.07–2.104 | 4.54 | 1 | 0.033 |
| Brazoria | 3 | 19 | 0.236 | -0.749–1.216 | 0.24 | 1 | 0.63 |
| Kirwin | 3 | 26 | 0.154 | -0.248–0.58 | 0.56 | 1 | 0.45 |
| Laguna Atascosa | 2 | 20 | 0.284 | -0.566–1.234 | 0.43 | 1 | 0.51 |
| Matagorda Island | 2 | 21 | 0.144 | -0.691–0.993 | 0.12 | 1 | 0.73 |
| McFaddin^c^ | 3 | 21 | -0.228 | -1.735–1.079 | 0.12 | 1 | 0.73 |
| San Bernard | 2 | 20 | -0.144 | -0.933–0.609 | 0.14 | 1 | 0.71 |
| Texas Point | 2 | 20 | -0.254 | -1.308–0.722 | 0.26 | 1 | 0.61 |
| Bufflehead (*Bucephala albeola*) |  |  |  |  |  |  |  |
| Crescent Lake | 2 | 9 | 0.372 | -0.375–2.763 | 0.74 | 1 | 0.39 |
| Goldeneyes (*Bucephala clangula*/*islandica*) |  |  |  |  |  |  |  |
| Kirwin | 3 | 28 | -0.238 | -0.754–0.195 | 1.12 | 1 | 0.29 |
| North Platte^d^ | 2 | 23 | 0.303 | -0.229–0.955 | 1.24 | 1 | 0.27 |
| Salt Plains | 2 | 21 | 0.551 | -0.388–1.649 | 1.36 | 1 | 0.24 |
| Common Merganser (*Mergus merganser*) |  |  |  |  |  |  |  |
| Kirwin | 2 | 28 | 0.208 | -0.191–0.647 | 1.05 | 1 | 0.31 |
| North Platte^d^ | 3 | 25 | 0.491 | 0.012–1.04 | 4.05 | 1 | 0.044 |
| Salt Plains | 3 | 24 | 1.395 | 0.263–3.039 | 6.10 | 1 | 0.014 |
| Ruddy Duck (*Oxyura jamaicensis*) |  |  |  |  |  |  |  |
| Bitter Lake | 3 | 9 | 0.063 | -0.698–0.843 | 0.03 | 1 | 0.87 |
| Crescent Lake | 1 | 8 | ^e^ | ^e^ | ^e^ | ^e^ | ^e^ |
| Quivira | 2 | 20 | -0.479 | -1.290–0.131 | 2.29 | 1 | 0.13 |
| Salt Plains | 3 | 24 | 0.049 | -0.685–0.773 | 0.02 | 1 | 0.89 |

^a^ Several new areas were added to the Anahuac surveys over time.

^b^ One new area was added to the Aransas surveys from October 1988 onward.

^c^ Several new areas were added to the McFaddin surveys over time.

^d^ One new area was added to the North Platte surveys from October 1991 onward.

^e^ No regression performed due to zero variation in the dependent variable (i.e., peak month).
