# Supplementary material for: Changes in waterfowl migration phenologies in central North America: Implications for future waterfowl conservation
Source: PLoS One. 2022 May 18;17(5):e0266785. doi: 10.1371/journal.pone.0266785 (PMC9116660; doi:10.1371/journal.pone.0266785)
Supplement: S1 Text — (DOCX) [file pone.0266785.s005.docx]

**S1 Text. Further details on the choice of phenological parameter.**

Given that our survey data are truncated to 3 months for each migration period (October–December and January–March), it is important that the phenological parameter used is robust against truncated distributions. Peak abundance month fulfills this requirement, as long as the distribution of monthly averages is unimodal or the highest peak occurs within the defined spring or autumn migration periods. In our case, this would likely be true for most refuges and species. Possible exceptions were early fall migrants and late spring migrants such as Blue-winged Teal (*Spatula discors*) and Cinnamon Teal (*S. cyanoptera*) throughout the included portion of the Central Flyway [31], other species at northern refuges, and refuges that serve as wintering locations where abundance generally exhibit a single peak in December or January.

However, refuges serving as wintering locations are of little concern as bird numbers tend to gradually increase and decrease at such locations, resulting in unimodal distributions. Late spring peaks or early autumn peaks could potentially present a more salient problem. However, because we treated peak month as an ordered categorical variable, as long as the highest peak within the defined migration periods occur in March for the spring season and October for the autumn season, the fact that the true peak occurs after March or before October does not invalidate or affect the interpretation of the results. Cases like these merely change the interpretation of the March and October categories to mean “in or after March” and “in or before October”, respectively. For example, the possible outcome categories for the fall migration period change from “peak occurred in October”, “peak occurred in November”, and “peak occurred in December” to “peak occurred in or before October”, “peak occurred in November”, and “peak occurred in December”. As there is no change in the ordering of these new categories, the analyses and interpretation of the results remain unchanged.

Further, an evaluation of all the refuges in our study offering survey data for 1 or more months prior to October (*n* = 18) or following March (*n* = 7) across all species and years revealed that bimodality with the highest peak outside the defined periods, where the peak within the defined migration season did not occur in the month closest to the true peak occurred in only 7 cases for autumn (*n* = 174) and 1 case for spring (*n* = 72). Thus, we were confident that our results did not overestimate the number of phenological shifts.
